# Dynamic brain network states in human generalized spike-wave discharges

**DOI:** 10.1093/brain/awy223

**Published:** 2018-08-28

**Authors:** Chayanin Tangwiriyasakul, Suejen Perani, Maria Centeno, Siti Nurbaya Yaakub, Eugenio Abela, David W Carmichael, Mark P Richardson

**Affiliations:** 1 Department of Basic and Clinical Neuroscience, Institute of Psychiatry Psychology and Neuroscience, King’s College London, London, UK; 2 Developmental Imaging and Biophysics Section, Developmental Neurosciences Program, UCL Great Ormond Street Institute of Child Health, London, UK; 3 King’s College Hospital, London, UK

**Keywords:** idiopathic/genetic generalized epilepsy, generalized spike-wave, EEG-functional MRI, functional brain network, pre-ictal

## Abstract

Generalized spike-wave discharges in idiopathic generalized epilepsy are conventionally assumed to have abrupt onset and offset. However, in rodent models, discharges emerge during a dynamic evolution of brain network states, extending several seconds before and after the discharge. In human idiopathic generalized epilepsy, simultaneous EEG and functional MRI shows cortical regions may be active before discharges, and network connectivity around discharges may not be normal. Here, in human idiopathic generalized epilepsy, we investigated whether generalized spike-wave discharges emerge during a dynamic evolution of brain network states. Using EEG-functional MRI, we studied 43 patients and 34 healthy control subjects. We obtained 95 discharges from 20 patients. We compared data from patients with discharges with data from patients without discharges and healthy controls. Changes in MRI (blood oxygenation level-dependent) signal amplitude in discharge epochs were observed only at and after EEG onset, involving a sequence of parietal and frontal cortical regions then thalamus (*P* < 0.01, across all regions and measurement time points). Examining MRI signal phase synchrony as a measure of functional connectivity between each pair of 90 brain regions, we found significant connections (*P* < 0.01, across all connections and measurement time points) involving frontal, parietal and occipital cortex during discharges, and for 20 s after EEG offset. This network prominent during discharges showed significantly low synchrony (below 99% confidence interval for synchrony in this network in non-discharge epochs in patients) from 16 s to 10 s before discharges, then ramped up steeply to a significantly high level of synchrony 2 s before discharge onset. Significant connections were seen in a sensorimotor network in the minute before discharge onset. This network also showed elevated synchrony in patients without discharges compared to healthy controls (*P* = 0.004). During 6 s prior to discharges, additional significant connections to this sensorimotor network were observed, involving prefrontal and precuneus regions. In healthy subjects, significant connections involved a posterior cortical network. In patients with discharges, this posterior network showed significantly low synchrony during the minute prior to discharge onset. In patients without discharges, this network showed the same level of synchrony as in healthy controls. Our findings suggest persistently high sensorimotor network synchrony, coupled with transiently (at least 1 min) low posterior network synchrony, may be a state predisposing to generalized spike-wave discharge onset. Our findings also show that EEG onset and associated MRI signal amplitude change is embedded in a considerably longer period of evolving brain network states before and after discharge events.

## Teaser

Generalised spike-wave discharges (GSW) in idiopathic generalised epilepsy (IGE) appear to have abrupt onset on EEG. However, in rodent models, GSW emerge during evolving brain network states. Using EEG-fMRI, Tangwiriyasakul *et al*. reveal that GSW onset in human IGE, as in rodent models, emerges during evolving brain network states.

## Introduction

Idiopathic generalized epilepsy (IGE) is typically subdivided into the clinical syndromes of childhood absence epilepsy (CAE), juvenile absence epilepsy (JAE), juvenile myoclonic epilepsy (JME) and generalized tonic-clonic seizures only. These syndromes differ in age of onset and relative occurrence of the characteristic seizure types (typical absence, myoclonus and tonic-clonic). Despite differences, these IGE syndromes share common features, especially the occurrence of paroxysmal generalized spike-wave discharges (GSW) seen with EEG, normal conventional clinical neuroimaging, and cognitive and motor functions typically within the normal range. Moreover, in families in which IGE is inherited, different family members may have different IGE syndromes, suggesting substantial genetic and mechanistic overlap (Helbig *et al.*, 2009; de Kovel *et al.*, 2010). In some instances, the presumed causative genetic variant is known and is implicated in several IGE syndromes. For example, the same microdeletions have been found in several IGE syndromes: microdeletions in 15q13.3 in cases with CAE, JAE and JME; in 15q11.2 in CAE, JAE, JME and generalized tonic-clonic seizures only; and in 16p13.11 in CAE and generalized tonic-clonic seizures only (Helbig *et al.*, 2009; de Kovel *et al.*, 2010). Furthermore, mutations in *CACNA1H*, which encodes for the T-type calcium channel Ca_v_3.2 strongly implicated in the generation of GSW, have been found in patients with CAE, JAE and JME (Chen *et al.*, 2003; Heron *et al.*, 2007; Klassen *et al.*, 2011). Based on this phenotypic and genetic commonality, this study is based on an assumption that there are shared pathophysiological features and important shared mechanisms across these four IGE syndromes.

GSW has been extensively studied in experimental models, with much progress made in elucidating underlying mechanisms (Crunelli and Leresche, 2002; Blumenfeld, 2005). Broadly, abnormalities of the parameters of interaction between neurons in cortex, thalamic reticular nucleus and thalamic specific nuclei, in part involving T-type calcium currents, give rise to GSW (Crunelli and Leresche, 2002; Blumenfeld, 2005). In two much-studied rat models of inherited GSW, Wistar Albino Glaxo from Rijswijk (WAG/Rijk) and Genetic Absence Epilepsy Rat from Strasbourg (GAERS), GSW onset is driven by a focal cortical region, which rapidly engages thalamocortical circuits (Meeren *et al.*, 2002; Polack *et al.*, 2007). Relevantly, pathogenic variation in CACNA1H has been implicated in GAERS rats (Powell *et al.*, 2009). Local field potential recordings in multiple regions of cortex and thalamus in WAG/Rijk rats has revealed peri-GSW dynamic evolution of brain network connectivity, strongly suggesting that GSWs do not have abrupt onset and are not primarily generalized, but emerge from a pro-ictal (seizure-permissive) network state (reviewed in Luttjohann and van Luijtelaar, 2015). Characteristic features of regional activity and inter-regional connectivity in a pre-ictal period lasting more than a second prior to GSW onset can be detected and used to predict GSW onset in WAG/Rijk rats with above-chance probability (Maksimenko *et al.*, 2017). The possibility that there is a peri-GSW dynamic evolution of brain network states has not been examined in human IGE.

Elucidating the brain network mechanisms underlying GSW in human IGE has been greatly advanced through simultaneous EEG-functional MRI (Aghakhani *et al.*, 2004; Gotman *et al.*, 2005; Hamandi *et al.*, 2006; Moeller *et al.*, 2008*a*, 2011*a*; Vaudano *et al.*, 2009; Carney *et al.*, 2010; Benuzzi *et al.*, 2012; Masterton *et al.*, 2013; Pugnaghi *et al.*, 2014; Guo *et al.*, 2016). Broadly, these studies have shown that during a GSW event, the blood oxygenation level-dependent (BOLD) functional MRI signal is decreased in a network of cortical regions and increased in thalamus, and that this network seems similar across different IGE syndromes. The cortical regions involved in GSW are similar in distribution to the so-called default mode network (DMN) (Fox *et al.*, 2005). Several EEG-functional MRI studies have attempted to determine whether specific brain regions show changes in activity immediately before the onset of GSW (Moeller *et al.*, 2008*a*; Bai *et al.*, 2010; Benuzzi *et al.*, 2012; Masterton *et al.*, 2013; Guo *et al.*, 2016), potentially being focal driving nodes as seen in rodent GSW models. These EEG-functional MRI studies have inconsistently found precuneus, prefrontal and parietal cortical regions to be active prior to GSW.

The EEG-functional MRI studies mentioned above have mostly not explicitly modelled the network connections between different brain regions. Investigation of functional brain networks, using EEG or functional MRI, has begun to cast light on mechanisms of human IGE. Using EEG to study IGE patients, network connectivity was shown to be globally increased in the low alpha band (Chowdhury *et al.*, 2014); in a modelling study, it was shown that this specific observed connectivity abnormality was inherently ictogenic (Petkov *et al.*, 2014). Using functional MRI to study functional brain networks in IGE, one study found no change in functional MRI functional connectivity during GSW-free periods between regions involved in the GSW network (Moeller *et al.*, 2011*b*), whereas other studies without EEG monitoring during functional MRI data collection found reduced functional connectivity between medial prefrontal cortex and precuneus (McGill *et al.*, 2012) and reduced connectivity between these regions and thalamus (Kim *et al.*, 2014).

In summary, the above evidence suggests that, in rats, GSW onset emerges from a specific functional network state, which might be regarded as a facilitatory or ‘pro-ictal’ network state, and is driven by localized regions before engaging wider thalamocortical circuits. In human IGE, there is incomplete evidence to suggest certain regions are active before GSW and may drive GSW; and incomplete evidence that specific network connections may be altered around the time of GSW. In this study, we set out to provide a comprehensive account of regional activity in the brain and brain network connectivity before, during, and after GSW, at the highest possible time-resolution using EEG-functional MRI; and to compare peri-GSW brain network activity with the brain state remote from GSW events. We tested the hypothesis that in human IGE, as in rodent models, GSW onset emerges from a specific functional network state and evolves through a sequence of network states.

## Materials and methods

The methods are summarized in Supplementary Fig. 1.

### Participants

We acquired data from 43 patients with IGE, studied either at the Institute of Psychiatry Psychology and Neuroscience, King’s College London (KCL) or the UCL Great Ormond Street Institute of Child Health (UCL). We acquired data from 21 patients with JME or generalized tonic-clonic seizures only at KCL [mean age ± standard deviation (SD): 24.2 ± 8.5 years], and 22 patients diagnosed with CAE or JAE at UCL (mean age ± SD: 9.6 ± 2.9 years). A total of 34 healthy age-matched healthy controls were studied: 18 at KCL (mean age ± SD: 23.9 ± 3.8 years), and 16 at UCL (mean age ± SD: 9.6 ± 2.4 years) (Table 1). Patients were recruited through first seizure clinics and EEG departments in several hospitals across London. Healthy controls were recruited by advertisements in our institutions. Participants were excluded if they had neurological diagnoses other than epilepsy, or a history of drug or alcohol misuse. Additional exclusion criteria for the healthy control subjects were personal history of seizures or family history of epilepsy. The study was approved by the Riverside Research Ethics Committee (12/LO/2006) and the London-Surrey Border Research Ethics Committee approved the acquisition of paediatric healthy controls (11/LO/1421). Written informed consent was obtained from all participants according to the Declaration of Helsinki (2013). A parent or the nominated legal carer gave written informed consent on behalf of the participant if below the age of 18.

**Table 1:** Summary demographics of patients and healthy controls

			Mann-Whitney	*P*-value	χ^2^	*P*-value
	**All patients**	**All controls**				
Subjects, *n*	43	34	-	-	0.219	0.639
Female, *n*	23	20				
Age, median (max, min)	14 (40, 5)	18.5 (34, 6)	672	0.548	-	-
	**Child patients**	**Child controls**				
Subjects, *n*	22	16	-	-	2.88	0.09
Female, *n*	9	11				
Age, median (max, min)	9 (16, 5)	9 (14, 6)	172	0.905	-	-
	**Adult patients**	**Adult controls**				
Subjects, *n*	21	18	-	-	1.11	0.291
Female, *n*	14	9				
Age, median (max, min)	21 (40, 13)	23.5 (34, 17)	158	0.381	-	-
	**All patients with GSW**	**All patients without GSW**				
Subjects, *n*	20	23	-	-	1.08	0.298
Female, *n*	9	14				
Age, median (max, min)	10 (40, 5)	16 (40, 6)	202	0.502	-	-
	**Child patients with GSW**	**Adult patients with GSW**				
Subjects, *n*	12	8	-	-	1.16	0.28
Events, *n*, median (max, min)	3 (10, 1)	5 (13, 1)	42	0.641	-	-

Table describes the age and sex characteristics of the studied groups of subjects, and the number of GSW events in the patients. Mann-Whitney U-test and *P*-value are shown for the comparison of age or number of GSW events between groups. Chi-squared statistic and *P*-value is shown for the comparison of the proportion of male and female subjects in each group or for the proportion of subjects in each group who had GSW during scanning or did not have GSW. Note none of the comparisons are significantly different, suggesting groups did not differ on these characteristics.

**Table 2:** Details of patients

Subject	Age	Gender	Syndrome	Total GSW events recorded	GSW events included in analysis	Mean GSW duration, s (SD)	AEDs
Pat-A1	21	Male	GTCSO	12	8	1.34 (0.41)	None
Pat-A2	16	Female	JME	0	0	–	None
Pat-A3	13	Female	JME	0	0	–	None
Pat-A4	20	Female	JME	0	0	–	None
Pat-A5	35	Female	JME	4	4	3.73 (0.62)	None
Pat-A6	15	Female	JME	0	0	–	None
Pat-A7	17	Female	GTCSO	0	0	–	None
Pat-A8	26	Female	JME	2	2	4.23 (1.17)	None
Pat-A9	25	Female	GTCSO	0	0	–	None
Pat-A10	31	Female	JME	6	5	1.42 (0.55)	None
Pat-A11	16	Female	GTCSO	0	0	–	None
Pat-A12	20	Male	JME	0	0	–	None
Pat-A13	19	Female	JME	14	13	1.11 (0.49)	None
Pat-A14	21	Male	JME	0	0	–	VPA
Pat-A15	22	Female	JME	1	1	0.61	LMT, LEV
Pat-A16	40	Male	JME	0	0	–	VPA
Pat-A17	39	Male	GTCSO	0	0	–	LEV
Pat-A18	22	Female	JME	0	0	–	LMT
Pat-A19	30	Female	JME	6	5	4.70 (6.68)	VPA, LEV
Pat-A20	40	Male	JME	7	5	1.26 (0.55)	VPA
Pat-A21	20	Male	JME	0	0	–	VPA, LEV
Pat-K1	14	Male	JAE	12	10	7.33 (9.26)	None
Pat-K2	10	Male	JAE	7	6	5.11 (3.68)	None
Pat-K3	8	Male	CAE	2	1	5.76	None
Pat-K4	16	Female	JAE	2	0	–	None
Pat-K5	7	Male	CAE	3	3	13.40 (4.84)	None
Pat-K6	9	Male	CAE	2	2	11.61 (0.11)	None
Pat-K7	5	Male	CAE	3	3	10.07 (5.64)	None
Pat-K8	6	Male	CAE	1	0	–	None
Pat-K9	6	Male	CAE	0	0	–	None
Pat-K10	13	Female	JAE	0	0	–	None
Pat-K11	10	Male	CAE	0	0	–	None
Pat-K12	9	Female	CAE	0	0	–	None
Pat-K13	13	Female	JAE	0	0	–	None
Pat-K14	9	Female	CAE	4	2	10.71 (1.03)	LMT, LEV
Pat-K15	9	Female	CAE	10	7	11.39 (3.30)	LMT
Pat-K16	10	Male	CAE	5	3	6.45 (3.51)	LMT, LEV
Pat-K17	10	Male	CAE	2	2	1.02 (0.42)	ETX
Pat-K18	8	Male	CAE	8	6	5.91 (3.07)	VPA, ETX
Pat-K19	7	Female	CAE	7	7	0.66 (0.19)	LMT, VPA
Pat-K20	8	Male	CAE	0	0	–	VPA, ETX
Pat-K21	13	Female	CAE	0	0	–	VPA
Pat-K22	12	Female	JAE	0	0	1.34 (0.41)	ETX, TPM

Age, sex, IGE syndrome, number of GSW events during scanning and antiepileptic drugs (AEDs) taken by each patient included in the study. ETX = ethosuximide; GTCSO = generalized tonic-clonic seizures only; LEV = levetiracetam; LMT = lamotrigine; VPA = valproic acid.

### Data acquisition and preprocessing

Participants underwent two runs of resting state functional MRI acquisition combined with EEG. We used a 3 T scanner at KCL (MR750, GE Healthcare) to acquire 300 echo-planar images per run (3.3 × 3.3 × 3.3 mm, field of view = 211 mm, repetition time = 2.160 s, echo time = 25 ms, flip angle 75°, 36 slices, slice thickness 2.5 mm). We used a 1.5 T scanner at UCL (Avanto, Siemens Healthcare), also acquiring 300 echo-planar images per run (3.3 × 3.3 × 4.0 mm, field of view = 210 mm, repetition time = 2.160 s, echo time = 30 ms, flip angle 75°, 30 slices, slice thickness 3 mm). EEG data were acquired with an MRI-compatible EEG cap containing 63 Ag/AgCl electrodes positioned according to the 10/10 system and referenced to FCz (Brain Cap MR, Brain Products). One additional electrode was positioned to record the ECG. Signals were sampled at 5000 Hz and EEG samples were synchronized with the MRI scanner clock. Impedances were kept under 10 kΩ. During scans, subjects were asked to rest with their eyes closed and stay awake.

Magnetic resonance gradient and pulse-related artefacts were removed off-line from the EEG recorded inside the MRI using template artefact subtraction (Allen *et al.*, 1998, 2000) implemented in Brain Analyzer (Brain Products). EEG was then downsampled to 250 Hz. GSW were identified by visual inspection and onset and offset marked for each session by S.P. and subsequently confirmed by CT. Because we were specifically interested in the dynamic evolution of brain networks before and after GSW, we only included GSW events with at least 30 functional MRI time points [30 repetition times (TRs) each of 2.16 s] before and after the GSW event.

To preprocess functional MRI data, we used SPM8 (r6313, ww.fil.ion.ucl.ac.uk/spm/) running on MATLAB (R2016b, The MathWorks Inc., Natick, MA) and the FIACH package (http://www.homepages.ucl.ac.uk/~ucjttie/FIACH.html) for R (3.2.2, https://www.r-project.org) (Tierney *et al.*, 2016). First, we converted DICOM data format into Nifti format. Second, the first four volumes of each session were deleted. We then realigned all images to the first remaining volume. Next, we applied the FIACH toolbox to correct for possible artefacts in the BOLD time series related to motion and other sources of noise. Subsequently, the data were spatially normalized into standard MNI space with 2 mm isotropic voxels. Next, all images were spatially smoothed using a Gaussian function of 8 mm full-width at half-maximum. BOLD signals were then bandpass filtered 0.04–0.07 Hz (Glerean *et al.*, 2012). This frequency range was chosen to minimize overlap between BOLD signal fluctuations of interest and possible artefacts from breathing and pulsation (Glerean *et al.*, 2012). We then parcellated the brain into 90 regions using the AAL atlas (Tzourio-Mazoyer *et al.*, 2002). Finally, we applied principal components analysis to the voxel time series within each region and extracted the first principal component to represent its activity (Friston *et al.*, 2006). The output of the data preprocessing stage was a 90 region × 296 TR matrix of regional time series (Supplementary Fig. 1).

### GSW related BOLD signal amplitude changes

To directly compare time-varying regional BOLD amplitude with subsequent time-varying network synchronization, we performed a model free amplitude-based analysis, based on the first principal component of the BOLD time series from each brain region, which was also used in the network analysis above. Here, we normalized these signals into a z-score scale. Then, we estimated the ensemble average z-score amplitude in each region at each time point (TR) across all GSW epochs. A similar statistical approach as in the phase-based analysis was undertaken as follows. First we plotted the histogram of z-score amplitude across all regions and all epochs. We then estimated the cut-off threshold at *P* = 0.99 (which was z-score = 0.246 for healthy controls, z-score = 0.207 for patients without GSW, and z-score = 0.350 for patients with GSW). All brain regions with BOLD amplitude exceeding the 1% threshold were plotted using a surface rendering tool. This series of images summarizes the time-varying BOLD amplitude in brain regions, across all GSW events, and can be directly compared with time-varying network synchronization.

### Analysis of brain network connectivity time-locked to GSW events using phase synchrony

We investigated the dynamic behaviour of large-scale brain networks by analysing phase synchrony of the BOLD signal between different brain regions. We took particular care to minimize the effects of noise within brain regions (using principal component analysis described above) and across networks (using tensor decomposition); and to align GSW onsets across GSW events.

The first step was to extract time-varying BOLD signal phase in a set of brain regions, time-locked to GSW onset, and construct a time-varying matrix of phase differences between each pair of regions.

For each GSW event, an epoch time-locked to the GSW was selected starting from 30 volumes (TR) prior and continuing 30 volumes after GSW onset on EEG. The volumes during the EEG GSW event from each epoch were resampled to either extend or compress the duration of each GSW to three TRs, to be similar to the mean GSW duration, so that the entire epoch for each GSW event was 63 volumes. The first volume of the GSW event was labelled TR0; 30 volumes prior to GSW labelled TR−30 to TR−1, volumes during GSW labelled TR0 to TR+2, and volumes after GSW labelled TR+3 to TR+30. Note that to align onset of GSW events across all epochs, we upsampled each entire 63 volume epoch from 0.4629 Hz (TR = 2.16 s) to 100 Hz. Subsequently, we realigned the data to the 0.01 s closest to GSW onset on the EEG, then downsampled each epoch back to the original sampling frequency 0.4629 Hz. Next, the Hilbert transform was applied to estimate instantaneous phase of the 90 regional time series. We then estimated the phase difference matrix at each time point by subtracting the phase angle between every pair of nodes, resulting in a series of 90 × 90 phase-difference matrices with zero diagonal, one matrix for each of the 63 volumes.

The second step was to extract from these phase difference matrices the most important spatiotemporal modes, and to normalize the phase synchrony estimates prior to statistical testing.

We aimed to derive a de-noised estimate of phase synchrony between brain nodes, which we assume will be composed of only the most important spatiotemporal modes for each subject. This was achieved by applying tensor decomposition to the time-varying phase difference matrices. This is similar to a principal component analysis in a higher dimensional space, to extract the main spatial-temporal modes of the data. We binarized the time-varying phase difference matrices, where if the phase difference was ≤π/6 the node pair was considered synchronous ‘in-phase’ and the corresponding binary matrix element equalled one, otherwise the node pair was considered asynchronous ‘out of phase’ and the element was zero (Ponce-Alvarez *et al.*, 2015). These binarized between node phase difference matrices were considered as phase synchrony matrices. The 90 × 90 × 63 binary matrix was decomposed into 12 rank-one tensors using non-negative tensor factorization (Lee and Seung, 1999; Gauvin *et al.*, 2014). The choice of 12 components is somewhat arbitrary but it is consistent with functional MRI resting state data that are typically broken down into 7 to 14 components (Allen *et al.*, 2014; Ponce-Alvarez *et al.*, 2015). Furthermore, 12 components explained ∼80% of the spatiotemporal variance in our data. Each component represented a weighted synchronization matrix. We then reconstructed the de-noised individual subject’s phase synchrony brain matrix by first multiplying each component with its time-varying strength and then adding all these components together. This reconstructed phase network was considered to have reduced spatiotemporal noise and reduced spurious components of network synchronization. Note that all diagonal elements in each matrix were set to zero. Next, we normalized every element inside the phase synchrony matrices by dividing by the maximum edge value from the surrogate network (a null model). A surrogate network was generated for each subject group. To generate a surrogate matrix, we first took all reconstructed phase synchrony matrices from all subjects in each group. Next, we randomized the elements inside each of these matrices while preserving the degree and strength distribution (Rubinov and Sporns, 2011). Finally, we averaged these randomized matrices to represent the surrogate matrix for each subject group.

### Statistical evaluation of connectivity at group level

To represent whole brain connectivity at the group level, in terms of phase synchrony, at each volume (TR) we averaged the phase synchrony matrices across all GSW events. To identify edges (connections) with significant synchrony, we then searched for the threshold to eliminate spurious edges representing non-significant phase synchrony. The threshold was estimated from the histogram of the normalized phase synchrony matrices. First, we established that the phase synchrony values across all of the edges from all 63 TRs time-locked to the GSW event were normally distributed. Then, we fitted a Gaussian function to the histogram. Using the probability of 0.99 from the fitted Gaussian, we identified the corresponding threshold value (0.889 in patient data time-locked to GSW), indicating the top 1% of most strongly synchronized edges across all 63 TRs.

### Visualizing nodes with the most influential connectivity

Having now identified the most significant network edges, we next calculated the eigenvector centrality of each node connected to the network of significant edges, as a way of easily visualizing the properties of the network composed of the strongest 1% of edges. High eigenvector centrality is found in nodes that have high influence in a network (Lohmann *et al.*, 2010). All network connections exceeding the top 1% threshold and all hub nodes connected to these edges were illustrated using a brain surface rendering tool, surfIce (downloaded from: https://www.nitrc.org/plugins/mwiki/index.php/surfice). This series of images summarizes the time-varying synchronization strength between brain regions and the strength of hub nodes, across all GSW events.

### Control analysis time-locked to random events in epochs without GSW in patients and in healthy controls

To examine whether the evolution of functional networks over time with respect to GSW events is specific to those events, we also examined epochs of patient data time-locked to random events in scanning runs in which GSW were not recorded. We also examined epochs of data from healthy controls time-locked to random events. For these data, we marked three random events for each scanning session in each individual. Any marker with insufficient duration of TR before or after the random event marker was removed from the analysis. Epochs consisting of 61 TRs time-locked to random events were extracted. Imaging data were parcellated into 90 regions and the first principal component of each region extracted, filtered from 0.04–0.07 Hz and the signal phase estimated using the Hilbert transform. Following this, phase-difference matrices were constructed and further processed exactly as above. The threshold to eliminate spurious edges in the networks was 0.909 for the patients without GSW and 0.917 for healthy controls.

### Network identification and synchrony measurements time-locked to GSW

In the analyses above we examined the activity of individual brain regions and the synchronization between them. We noted that the time-varying distribution of significant network connections and hubs tended to implicate three dominant brain networks (see ‘Results’ section for full description): a network characteristic of GSW events in IGE patients; a network characteristic of IGE patients between GSW events (hubs predominantly in central sensorimotor regions); and a network characteristic of healthy control subjects (hubs predominantly in posterior hemisphere regions). This observation motivated us to examine synchronization within these three heuristically defined networks.

We identified the three canonical networks as follows. First, for each epoch (63 TRs as above) we removed spurious edges from the phase synchrony matrices for each epoch (90 regions × 90 regions × 63 TRs, reconstructed from 12 components) using the previously estimated threshold (see ‘Statistical evaluation at group level’ section above). Note that this is different from the previous analysis, where we estimated the group average across all epochs prior to removing spurious edges. Second, we identified the time periods and subject group in which each network was dominant. The GSW network was identified during TR0 to TR+2 in patient epochs with GSW events. The sensorimotor network was identified during TR−10 to TR−2 in patient epochs with GSW events. The posterior network was identified during TR−20 to TR−10 in healthy control epochs with random events. Third, we identified the network nodes and edges for each network. For each network, during the relevant TR period, we estimated the grand average and standard deviation of node eigenvector centrality. We defined each network as the set of edges connecting nodes with eigenvector centrality >1 SD above the grand average during the relevant TR period. We applied these network templates to each TR in each epoch; a total level of synchrony in each network at each TR was estimated as the sum of the level of synchrony in all significant edges in the network, divided by the number of edges in the network, to obtain a normalized value that can be compared between epochs and between networks.

We then compared this dynamic network synchrony measure in the three networks between patients during GSW epochs, patients without GSW and healthy controls. To identify TRs where network synchrony was significantly high or low in patients during GSW epochs compared to epochs without GSW, we estimated the 99% confidence intervals (CIs) for network synchrony for each network, across all 61 TRs, in the patient group without GSW events, and compared the patients with GSW events and healthy control subjects with this 99% CI.

### Average network synchrony

In the previous analyses, we examined time-varying brain network synchrony, comparing between time-points and between groups, in epochs time-locked to GSW events and epochs time-locked to random events in subjects without GSW.

Here we compare the average synchrony in brain networks across the entire functional MRI runs between groups of subjects. We investigated the same three canonical networks as above. Similar steps as explained in the above section were repeated (except that we considered the whole time series), resulting in a de-noised synchronization matrix of 90 × 90 × 296 elements, where 296 is the number of TR in each run. Next, we then subsampled this matrix by including only the nodes of each of the three networks (GSW network, sensorimotor network, posterior network), resulting in a matrix of *m* × *m* × 296, where *m* is a number of nodes in each network. At each TR, we estimated the mean degree in each network and then averaged across all 296 time points. To avoid global variability between functional MRI runs, we used the normalized mean degree (which is the mean degree of each network divided by the mean degree of the whole brain when *m* = 90). Finally, we applied non-parametric ranking of covariance (Quade’s test) and Mann-Whitney test (corrected for age) to check for significant differences between groups, using a Bonferroni-corrected threshold of *P* < 0.01.

### Data availability

Researchers wishing to access the data reported here for further research should contact the corresponding author. Where not in conflict with constraints of the original ethical approval under which the data were collected, pseudo-anonymized data will be made available for appropriate collaborative research, subject to appropriate ethical approval being gained for such use.

## Results

Seventy-seven subjects were studied, 43 with IGE and 34 healthy control subjects. A total of 120 GSW epochs were recorded during functional MRI, but after excluding epochs with less than 30 TRs before and after the GSW event, 95 GSW epochs from 20 subjects with IGE were included (Table 1). These subjects comprised eight patients with JME or generalized tonic-clonic seizures with a median of five GSW epochs per subject (range 1–13), and 12 patients with CAE or JAE with a median of three GSW epochs per subject (range 1–10). A total of 97 random epochs in healthy controls and 96 random epochs in patient functional MRI runs without GSW were also analysed.

### GSW related BOLD signal amplitude changes

**Figure 1:**
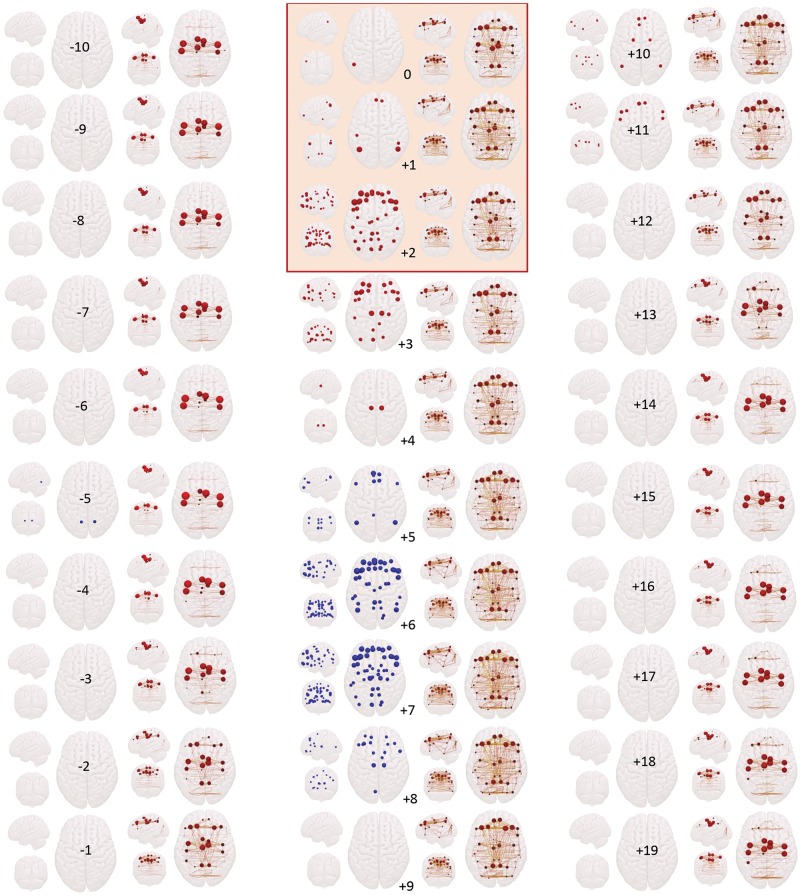
**Brain network evolution around GSW events (TR−10 to TR+19).** For each TR, there are two sets of three orthogonal ‘glass-brain’ projections (from left, front and top view). In the *left column* at each TR, we show significant increase and decrease in BOLD amplitude; red spheres are BOLD increases and blue spheres decreases. The radius of the sphere in the BOLD amplitude plots represents the magnitude of BOLD amplitude. In the *right column*, the lines are significant edges, and red spheres are hub nodes, where the radius of each sphere represents the eigenvector centrality value. The duration of the GSW event covers the period TR0 to TR+2 (red box). Note the reconfiguration and spread of the synchronization-based network prior to and at the emergence of GSW, in particular the gradual emergence of prefrontal and precuneus nodes from TR−3. Note also the successive increase and decrease of BOLD signal amplitude in all regions in which BOLD signal change is observed; and that BOLD amplitude changes tend to be ventral to hub nodes.

Figure 1 shows that the regions with 1% highest or lowest group-average BOLD signal amplitude were in regions associated with BOLD signal change in conventional general linear model (GLM) analyses, closely following the occurrence of GSW on EEG, during TR0 until TR+8. We observed that BOLD signal amplitude started to increase at TR0 over the left angular gyrus, extending to bilateral parietal and inferior medial frontal in TR+1, and rapidly involving bilateral cortical regions, particularly frontal, in TR+2. Subsequently at TR+3, the widespread increased BOLD diminished to parts of the frontal lobes, temporal lobes and motor cortex, while increased BOLD was observed in thalamus. At TR+4, the increased BOLD activity was only seen in the thalamus. Subsequently in TR+5, increased BOLD subsided, followed by decreased BOLD in similar brain regions as increased BOLD, and involving these regions in similar temporal sequence. In TR+5, decreased BOLD was observed in the frontal and parietal cortex, which became more extensive, and then waned as decreased BOLD in thalamus emerged. Note that changes in BOLD amplitude during GSW mostly implicated regions somewhat ventral to the main hubs of the GSW synchronization network.

As expected, in the group of patients with no GSW and in healthy controls, both time-locked to random events, the regions with 1% highest or lowest BOLD signal amplitude were observed with no relationship to the random events and in no meaningful anatomical relationship or temporal sequence (Supplementary Videos 1–3).

### Analysis of brain network connectivity time-locked to GSW events using phase synchrony

The significant network features time-locked to GSW events are illustrated in Fig. 1. Prior to the emergence of GSW, until TR−5, the 1% strongest connections consisted of a distributed set of transcallosal connections and connections between medial and lateral cortical regions in the same hemisphere, in frontal, parietal and occipital lobes. The most prominent hubs amongst these strongest connections were in dorsal regions of central sensorimotor cortex. This network was stable and persistent up to TR−5; subsequently, during TR−4 to TR−1, there was a gradual emergence of connections within and between dorsal prefrontal regions and precuneus, and anterior-posterior connections involving these nodes; sensorimotor hubs became less prominent, with the emergence of prefrontal and precuneus hubs. During the GSW, there was further emergence of widespread transcallosal and anterior-posterior connections and widely distributed prefrontal, central and precuneus hubs. This network pattern persisted until TR+11, following which there was gradual loss of prefrontal and precuneus hubs and re-emergence of a set of prominent sensorimotor hubs, as seen several TRs prior to GSW (see details of hub regions in Table 1).

### Control analysis time-locked to random events in epochs without GSW in patients and in healthy controls

Random-event epochs in healthy control subjects were dominated by a network that was highly stable, consisting of a distributed set of transcallosal connections and connections between medial and lateral cortical regions in the same hemisphere, in frontal, parietal and occipital lobes, with hubs mostly in posterior hemisphere regions. Random-event epochs without GSW events in patients were dominated by a relatively stable network similar to the healthy control subjects, sometimes switching to a network with prominent sensorimotor hubs, similar to the network observed prior to GSW. The dynamic changes in network edges and hubs are illustrated in Supplementary Videos 1–3.

### Time-varying synchrony within canonical brain networks

The analysis above of brain network synchrony time-locked to GSW events revealed two characteristic networks: a central sensorimotor network, prominent before and after GSWs (and prominent in epochs from patients without GSW events); and a network characteristic of the GSW event itself. The analysis above of brain network synchrony time-locked to random events in healthy control subjects and in patient epochs without GSW revealed a characteristic network in posterior regions. Here, we examine these three canonical networks.

**Figure 2:**
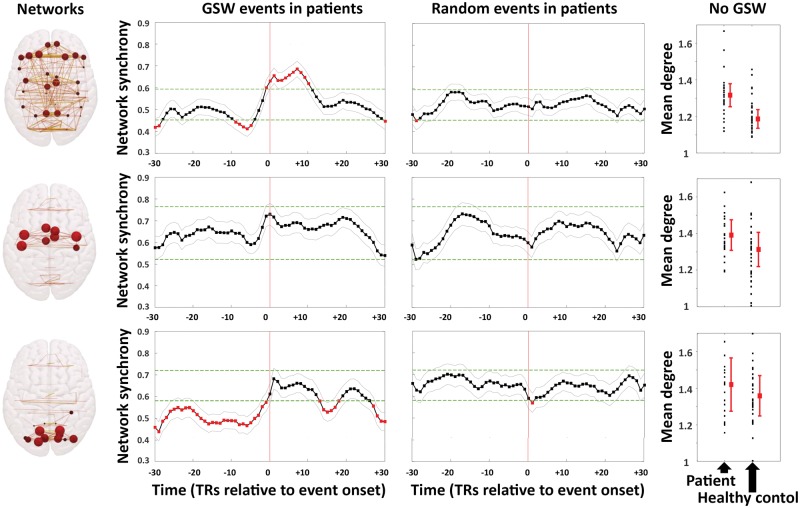
**Time course of normalized synchrony in three canonical brain networks before, during and after GSW events or random events.** We present three rows of data, one for each network: *top row*, GSW network; *middle row*, central sensorimotor network; *bottom row,* occipital network. Networks: cartoons of the distribution of network edges and hubs of each of these networks. Supplementary Table 2 shows the anatomical locations of nodes in each network. GSW events in patients: the group mean normalized network synchrony at each TR (± its standard error). The *x*-axis shows time indicated in TRs from TR−30 to TR+30 (one TR lasts 2.16 s). The *y*-axis is group mean normalized network synchrony. The vertical red line marks the GSW onset (TR = 0). The light green lines represent the 99% CI of the group mean normalized network synchrony, estimated from the 96 random event epochs from the functional MRI runs without GSW. Significantly high/low synchrony is highlighted in red. Random events in patients: control synchrony time courses for epochs time-locked to random events without GSW in patients. The vertical red line marks the random event onset (TR = 0). No GSW: average network synchrony (in terms of normalized mean degree) averaged within each network over the entire 296 TR functional MRI runs for all functional MRI runs without GSW in patients, and for healthy controls. The red square is the median, whiskers show 25th and 75th centiles; black squares are each individual subject. Note that average synchrony is higher in patients than controls in the GSW network and in the central sensorimotor network, over the entire 296 TR epochs without GSW events. Average synchrony in the occipital network does not differ between patients and healthy control subjects.

Figure 2 shows that during the GSW event and until TR+12 there is a significantly elevated level of synchrony in the GSW network, compared to the level of synchrony in the GSW network during epochs without GSW events. Prior to the GSW event, the level of synchrony in the GSW network is similar to the level of synchrony in this network in epochs without GSW events, except for a transient significant reduction in synchrony during TR−8 to −5, and then a rapid ramping-up of synchrony from TR−6 to GSW onset. In contrast, the level of synchrony in the GSW network in patients during epochs without GSW events remains relatively constant.

During epochs with GSW events, the occipital network shows significantly reduced synchrony throughout all of the peri-event epoch prior to the GSW event and for some of the time after the GSW event; during epochs without GSW, the synchrony in this network is consistently higher.

In contrast to the GSW and posterior networks, which showed significant peri-GSW changes in synchrony, during epochs with GSW events the level of synchrony in the central sensorimotor network increases only slightly during the GSW event, remaining similar to the level of synchrony in this central sensorimotor network in epochs in patients without GSW events.

### Average network synchrony over entire functional MRI runs without GSW

We compared the average level of synchrony (in terms of normalized mean degree) across entire functional MRI runs (296 TRs) in the three canonical networks, between functional MRI runs in IGE patients without GSW and healthy control subjects (Fig. 2). There was no significant difference between patients and healthy controls in the occipital network, but both the central sensorimotor network (*P* = 0.004) and the GSW network (*P* < 0.001) showed significantly higher average synchrony in the patients compared to controls, even in these runs without GSW events.

## Discussion

In this study, we tested the hypothesis that, in human IGE as in rodent models, GSW onset emerges from a specific functional network state and evolves through a sequence of network states. This hypothesis was confirmed: for the first time, we found prominent dynamic changes in brain networks in the peri-GSW period in human GSW. Moreover, we found that brain network features in people with IGE differ from healthy networks, even remote from GSW events.

For at least 1 min prior to GSW onset, strong hubs were seen in central sensorimotor regions; we also observed an increase in sensorimotor network synchrony in epochs from patients without GSW events. During the same 1 min prior to GSW onset, synchrony in the posterior network was significantly lower than in patient epochs without GSW. This combination of network states—persistently increased sensorimotor synchrony and transiently decreased posterior synchrony—may be a ‘pro-ictal’ (GSW-permissive) brain state. Approximately 6 s prior to GSW onset, prefrontal regions and precuneus became hub nodes, and synchronization between a widespread set of cortical hubs became prominent. Of note, this set of network hubs characteristic of GSW showed a significant linear increase in synchrony over the 10 s prior to GSW onset on EEG, suggesting that the gradual evolution of a set of sensorimotor network hubs to an ictal GSW network is a ‘pre-ictal’ brain state. At GSW onset there were prominent hubs in medial parietal cortex, and medial and lateral prefrontal cortex, most of which were relatively dorsal. A high level of synchrony persisted in the GSW network until about 20 s after GSW offset on EEG, thereafter the brain settled to the pre-GSW state, with strong hubs in central sensorimotor regions.

In addition to our analysis of time-varying network connectivity, we also evaluated a simple measure of average network synchrony over the entire functional MRI run. Average degree is total connectedness in the network, normalized by the number of network nodes, and hence is a very appealing measure to compare between subjects and between networks. We took this approach to be able to ask the simple question: on average over time, in this network in this subject, is network synchrony relatively high or low, compared to other subjects and other networks? This approach revealed that average synchrony is persistently higher in patients than controls in the GSW network and in the central sensorimotor network.

### Changes in BOLD amplitude related to GSW

We show a similar anatomical distribution of BOLD amplitude changes as described previously (Aghakhani *et al.*, 2004; Gotman *et al.*, 2005; Hamandi *et al.*, 2006; Moeller *et al.*, 2008*a*, *b*; Bai *et al.*, 2010; Carney *et al.*, 2010; Benuzzi *et al.*, 2012; Masterton *et al.*, 2013). Importantly, we show the temporal sequence in which BOLD signal amplitude changes in different regions, and, for the first time, we show how BOLD amplitude changes are a relatively brief component of a much longer-lasting peri-GSW series of changes in BOLD functional network states. Our data also show that BOLD signal in all regions followed a pattern of increased BOLD signal followed by decreases with variable time of onset in different regions, and did not support the notion of DMN deactivation.

EEG-functional MRI studies usually assume that the change in neuronal activity of interest occurs around the onset of GSW and terminates around offset of GSW, and fit a model of onset and offset convolved with a haemodynamic response function. Using such models, activation has typically been observed during GSW in thalamus and widespread deactivation in a distributed set of cortical regions approximating the so-called default mode network (DMN) (Aghakhani *et al.*, 2004; Gotman *et al.*, 2005; Hamandi *et al.*, 2006; Bai *et al.*, 2010). Some studies (Moeller *et al.*, 2008*a*; Carney *et al.*, 2010; Benuzzi *et al.*, 2012) have analysed activated and deactivated brain regions *post hoc*, which permits some insight regarding the temporal order of activity onset in different brain regions. These studies have suggested that BOLD signal change is detectable in cortical regions several seconds before GSW onset on EEG, implicating first onset variously in precuneus, parietal and prefrontal regions (Moeller *et al.*, 2008*a*; Carney *et al.*, 2010; Benuzzi *et al.*, 2012). Using a different approach, independent component analysis applied to peri-event BOLD data, prefrontal cortical regions were shown to have BOLD change several seconds ahead of GSW events (Masterton *et al.*, 2013). However, not all studies have found evidence of BOLD change prior to GSW (Moeller *et al.*, 2008*b*).

One previous study took a model-free approach, extracting epochs of BOLD data from multiple GSW events, time-locking all events to the onset of GSW, and then averaging across events, similar to our approach here, and revealed findings with similarities to ours (Bai *et al.*, 2010). This study found that all brain regions in which there was BOLD signal change in relation to GSW showed an increase in BOLD followed by a fall below the baseline, with similar haemodynamic response function-like time course in all regions, but the time of initial onset of BOLD increase was highly variable between regions: occurring up to 14 s before onset of GSW in some cortical regions (particularly medial prefrontal), during GSW in other cortical regions, and 10 s after GSW onset in thalamus. Therefore, the Bai *et al.* (2010) study found brain activity related to GSW began several seconds in advance of the GSW and extended several seconds after termination; and did not fit the concept of deactivation in DMN regions (Gotman *et al.*, 2005).

The same group of investigators recently reported a data-driven approach to identifying temporal ordering of onset of BOLD activity in different regions, related to GSW (Guo *et al.*, 2016). The first set of regions to show BOLD change involved DMN regions including medial frontal cortex; the second set of regions to become active involved more extensive neocortical regions in frontal and parietal lobes; the third set of regions to become active involved thalamus, primary sensorimotor and occipital regions. Their method to identify sets of regions, focussed entirely on BOLD amplitude rather than phase coupling in functional networks, was different from ours, but nonetheless the temporal order of involvement of brain regions has similarities.

### BOLD functional networks and underlying electrophysiological connectivity

We have studied functional networks using BOLD, with the assumption that functional connectivity measured with BOLD is meaningfully related to underlying electrophysiological functional connectivity (Schnitzler and Gross, 2005). However, BOLD signals fluctuate much more slowly owing to the effective low pass filter of the haemodynamic response and have a complex relationship with underlying electrophysiology, therefore the rationale for regarding functional networks based on BOLD signal phase as relevant for the interrogation of brain function may seem less clear. The analysis of phase synchronization in functional MRI BOLD signals has provided valuable insights into functional brain networks during performance of visual or motor-related tasks (Laird *et al.*, 2002; Glerean *et al.*, 2012). Importantly, there is electrophysiological evidence showing correlation between BOLD fluctuations and modulation of gamma frequency local field potentials (Nir *et al.*, 2007, 2008; He *et al.*, 2008; Shmuel and Leopold, 2008; Miller *et al.*, 2009), with some evidence that this associates with local neuronal firing rate (Nir *et al.*, 2007, 2008; Shmuel and Leopold, 2008). Furthermore, modelling studies have demonstrated that many features of resting state functional MRI BOLD networks can be replicated by assuming a linear relationship between neuronal firing rate at each node and phase angle of local fields, a standard Balloon-Windkessel model to estimate BOLD from the firing rate, and connectivity determined by observed diffusion tensor imaging (Cabral *et al.*, 2011; Vasa *et al.*, 2015). Therefore, if there is sufficient information transfer between two (or more) distinct regions of the brain, they will influence each other (Strogatz and Stewart, 1993) and this will eventually tune their spiking rates to be the same, and subsequently shift the phase angle of the BOLD signals to be similar. Therefore we argue that BOLD phase synchronization has a meaningful relationship to underlying electrophysiological interactions within functional brain networks.

### Key components of functional networks in idiopathic generalized epilepsy

We characterized functional networks particularly in terms of highly connected hub nodes. As a measure of ‘hubness’, we used eigenvector centrality—this measure quantifies how strongly a highly connected node is connected to other highly connected nodes. Therefore a node with high eigenvector centrality has high influence over the entire network, not just high influence over its connected neighbours.

We found a consistent and temporally stable set of highly connected nodes in bilateral sensorimotor areas which appeared characteristic of patients with IGE, suggesting a possible role for the sensorimotor network in predisposition to GSW. This finding is consistent with increased task-related functional connectivity to motor regions found in a previous study using functional MRI in JME (Vollmar *et al.*, 2011) supporting the potential role of motor network hyperconnectivity in IGE patients as a baseline endophenotype.

We found that about 6 s before GSW onset, additional nodes (prefrontal and precuneus) emerged as additional hubs connected with the motor network (see details of anatomical location of hub nodes in Supplementary Table 1). We propose that these prefrontal and precuneus nodes are a key component of triggering GSW events. Medial prefrontal cortex has been implicated as a region of structural abnormality in morphological studies of JME (O’Muircheartaigh *et al.*, 2011; Cao *et al.*, 2013). Notwithstanding the different timescale of investigation, a frontal onset of the initial spike of GSW has been identified in EEG and MEG (Holmes *et al.*, 2004; Stefan *et al.*, 2009; Tenney *et al.*, 2013). It is also notable that in a model-free analysis of BOLD amplitude changes reported by another group (Bai *et al.*, 2010), there is early BOLD change in medial prefrontal regions. Additionally, our previously published ictal onset modelling study based on EEG functional networks from IGE patients suggested GSW events could be driven most easily from a prefrontal node (Schmidt *et al.*, 2014*b*). We speculate that the engagement of prefrontal regions by a hypersynchronized motor network is a key causative factor in initiating a GSW event.

### Strengths and weaknesses of our study

We believe our sample of patients and GSW events is sufficient to confidently make inferences on our data. The number of GSW events is unbalanced across subjects, but we undertook an exploratory analysis in which two GSW events from each of 18 subjects, and one GSW event from the remaining two subjects, were included in the analysis and essentially identical findings were made. We collected data from children with CAE and JAE on a 1.5 T scanner, and data from adults with JME and generalized tonic-clonic seizures only on a 3 T scanner, and did not separate patients by age or syndrome or scanner type. However, in a further exploratory analysis in which we separately analysed the KCL data and UCL data, the findings were similar; in particular, the sequence of network changes prior to GSW was essentially identical, with a sensorimotor network prominent prior to GSW, extending over the 6 s prior to GSW to include prefrontal and precuneus nodes (Supplementary Fig. 3).

To study functional brain networks, connections are conventionally estimated using temporal correlations between nodes, computed over sliding time windows. Recently, phase synchronization (which, in contrast to analysis over sliding windows, can preserve the temporal resolution of the data) was introduced to construct a correlation matrix representing the brain network at the instantaneous moment. A group of two (or more) brain nodes with similar phase angle are assumed to have coordinated information transfer among the nodes (Strogatz and Stewart, 1993). Phase locking factor is a standard measure used to estimate a level of phase synchrony in M/EEG data (Roach and Mathalon, 2008; Schmidt *et al.*, 2014*a*). Unlike EEG or MEG, phase-related analysis is not widely applied to functional MRI data, although a few examples can be seen recently (Laird *et al.*, 2002; Glerean *et al.*, 2012).

Our ability to make these observations was enhanced by taking considerable steps to reduce noise in our data. Motion and physiological noise were optimally reduced using FIACH; regional time series data were de-noised using principle components analysis and tensor decomposition. Furthermore, using instantaneous phase information increased temporal resolution of this analysis as compared to a conventional sliding window correlation analysis using BOLD amplitudes. Moreover, we adopted stringent statistical thresholds, *P* < 0.01 across all edges in all TRs for the functional connectivity analysis, and *P* < 0.01 across all regions in all TRs for the BOLD amplitude analysis.

### Pathophysiological interpretation of our findings

As we have already discussed, in the rodent GSW models GAERS and WAG/Rijk, GSW onset is driven by a focal cortical region, which rapidly engages thalamocortical circuits (Meeren *et al.*, 2002; Polack *et al.*, 2007). Although our data do not provide sufficient spatial or temporal resolution to provide strong evidence, we speculate that prefrontal and precuneus nodes that emerge in the seconds prior to GSW onset might represent driving regions in human GSW. Notably, the WAG/Rijk model has been studied in detail over the peri-GSW state, revealing a cascade of changes prior to GSW onset in cortico-thalamo-cortical networks, that predispose to GSW onset (Luttjohann and van Luijtelaar, 2015; Maksimenko *et al.*, 2017). We speculate that our observations of dynamic changes in network activity in the seconds prior to GSW in human IGE reflect a similar cascade of changes in cortico-thalamo-cortical networks.

## Conclusion

Our findings here have highlighted that the underlying mechanism of generation of GSW involves a multistage process, including a pro-ictal network state lasting at least 1 min, and a pre-ictal network state lasting several seconds prior to GSW observed with EEG. Our findings are in keeping with the evidence from rodent models of GSW, that there is a pre-GSW alteration in regional activity and functional connectivity between regions, and greatly increase our understanding of the phenomena occurring in the peri-GSW period in human IGE.

## Supplementary Material

Supplementary Material

Supplementary Videos

## Abbreviations

BOLD: blood oxygenation level-dependent
CAE: childhood absence epilepsy
GSW: generalized spike-wave discharges
IGE: idiopathic generalized epilepsy
JAE: juvenile absence epilepsy
JME: juvenile myoclonic epilepsy
TR: repetition time
