# Supplementary material for: Dynamic brain network states in human generalized spike-wave discharges
Source: Brain. 2018 Aug 28;141(10):2981–94. doi: 10.1093/brain/awy223 (PMC6158757; doi:10.1093/brain/awy223)
Supplement: Supplementary Material [file awy223_supplementary_material.pdf]

Supplementary material

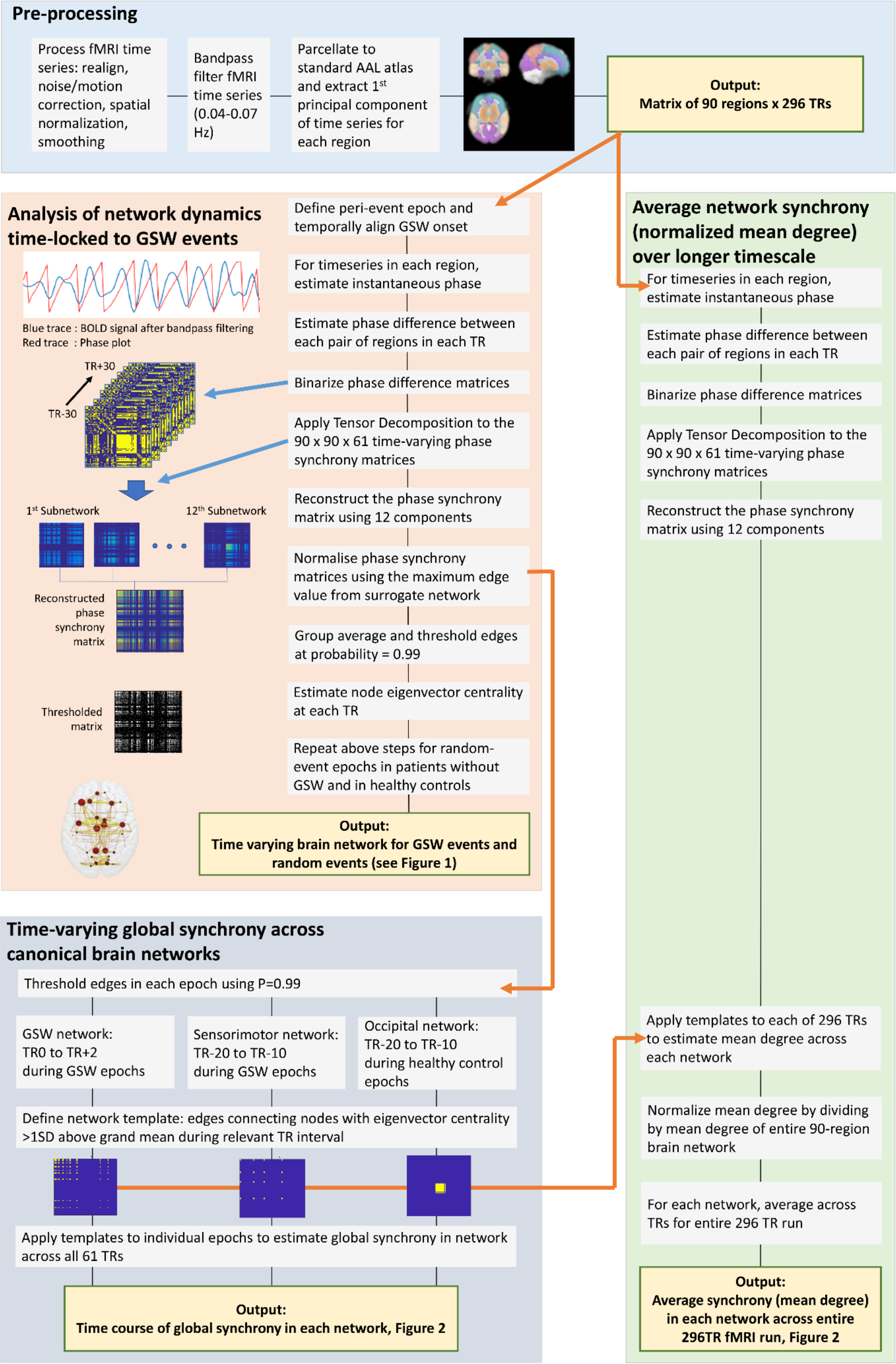

Figure 1: Analysis pipeline.

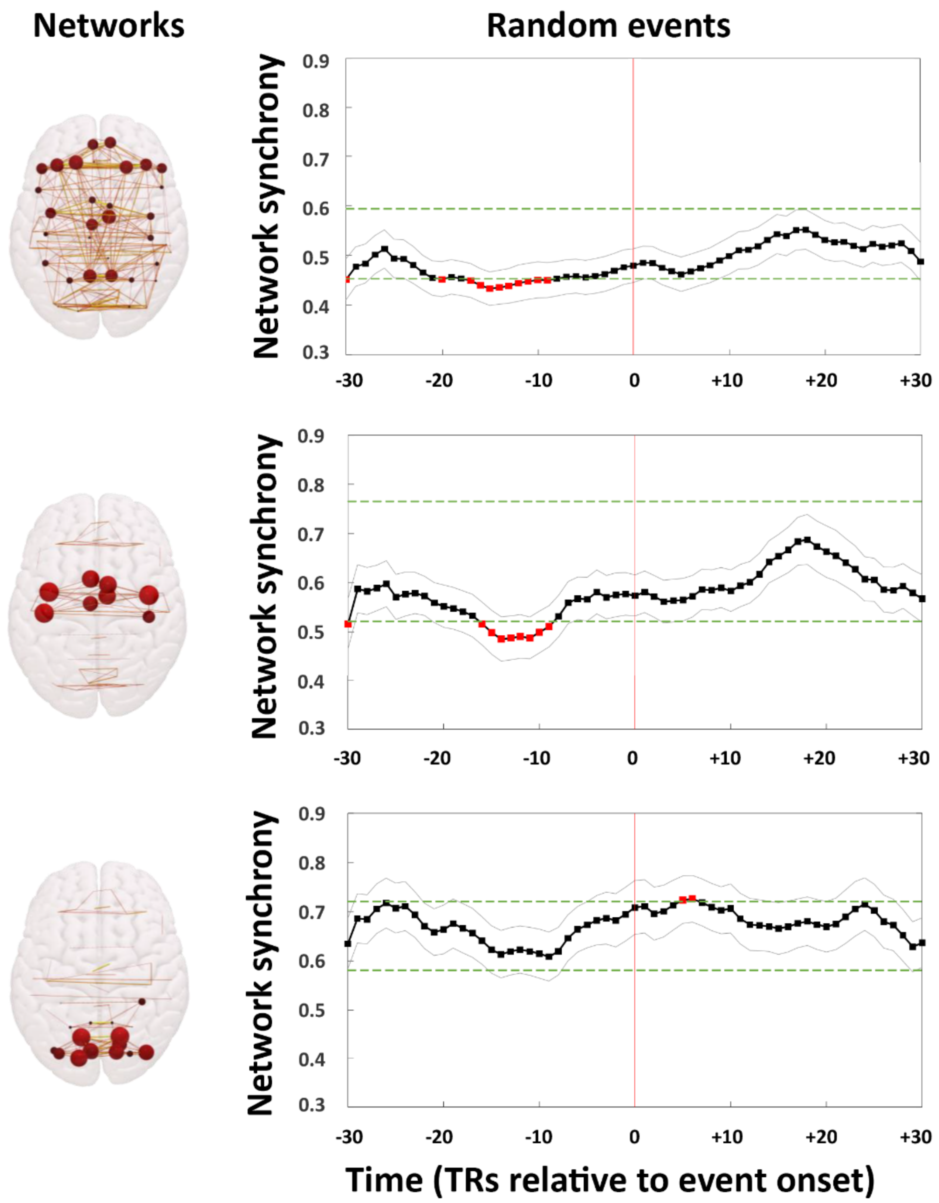

Figure 2: Time course of normalised synchrony in three canonical brain networks before, during and after random events in healthy controls. We present three rows of data, one for each network: top row GSW network; middle row central sensorimotor network; bottom row occipital network. The left column shows cartoons of the distribution of network edges and hubs of each of these networks. The right column shows the group mean normalised network synchrony at each TR (+/- its standard error). The x-axis shows time indicated in TRs from TR-30 to TR+30 (1 TR lasts 2.16 seconds). The y-axis is group mean normalised network synchrony. The vertical red line marks the random event onset (TR=0). The light green lines represent the 99% confidence interval of the group mean normalised network synchrony, estimated from the 96 random event epochs from the fMRI runs in patients without GSW. Significantly high/low synchrony is highlighted in red.

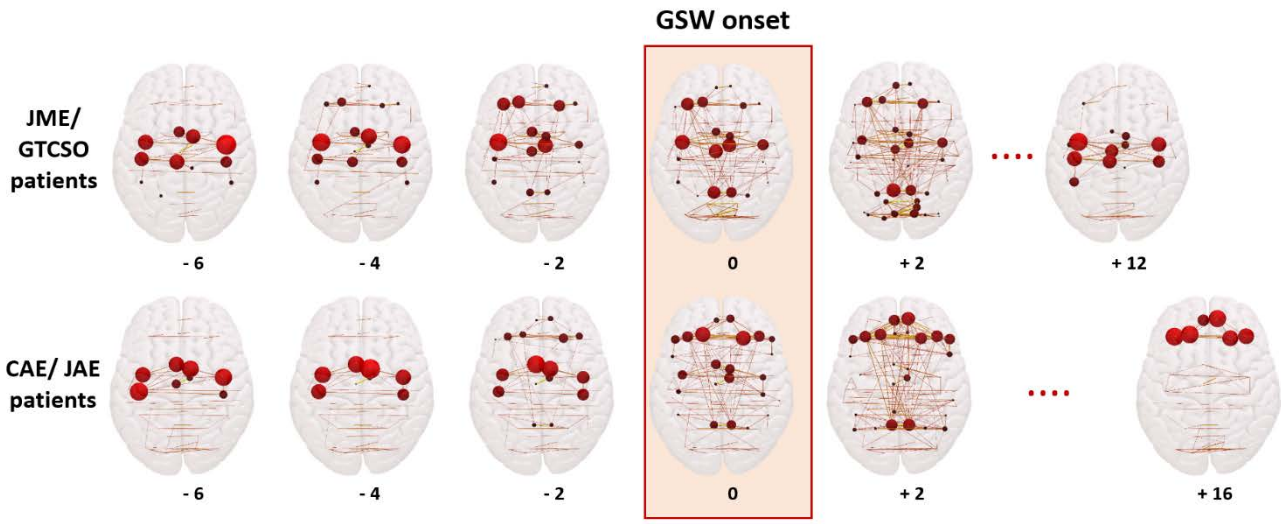

Figure 3. Posthoc analysis of JME/GTCSO and CAE/JAE subgroups. We sought in our analysis in the main manuscript to identify effects common across the IGE syndromes. However, it is of great interest to examine the syndromes separately, especially since adults with JME and GTCSO were scanned at 3T and children with CAE and JAE at 1.5T. Here, we split the subjects into these two groups, but otherwise the data analysis is identical to the main manuscript. Note that the effects prior to GSW onset are extremely similar: in both groups, prominent hub nodes are seen in central sensorimotor regions; prior to GSW onset there is the emergence of additional prefrontal and precuneus hubs. At TR0, there are prominent anterior-posterior connections at the GSW onset in both groups. Following GSW offset, the further evolution of network hubs differs between the groups, which may reflect differences in GSW durations.

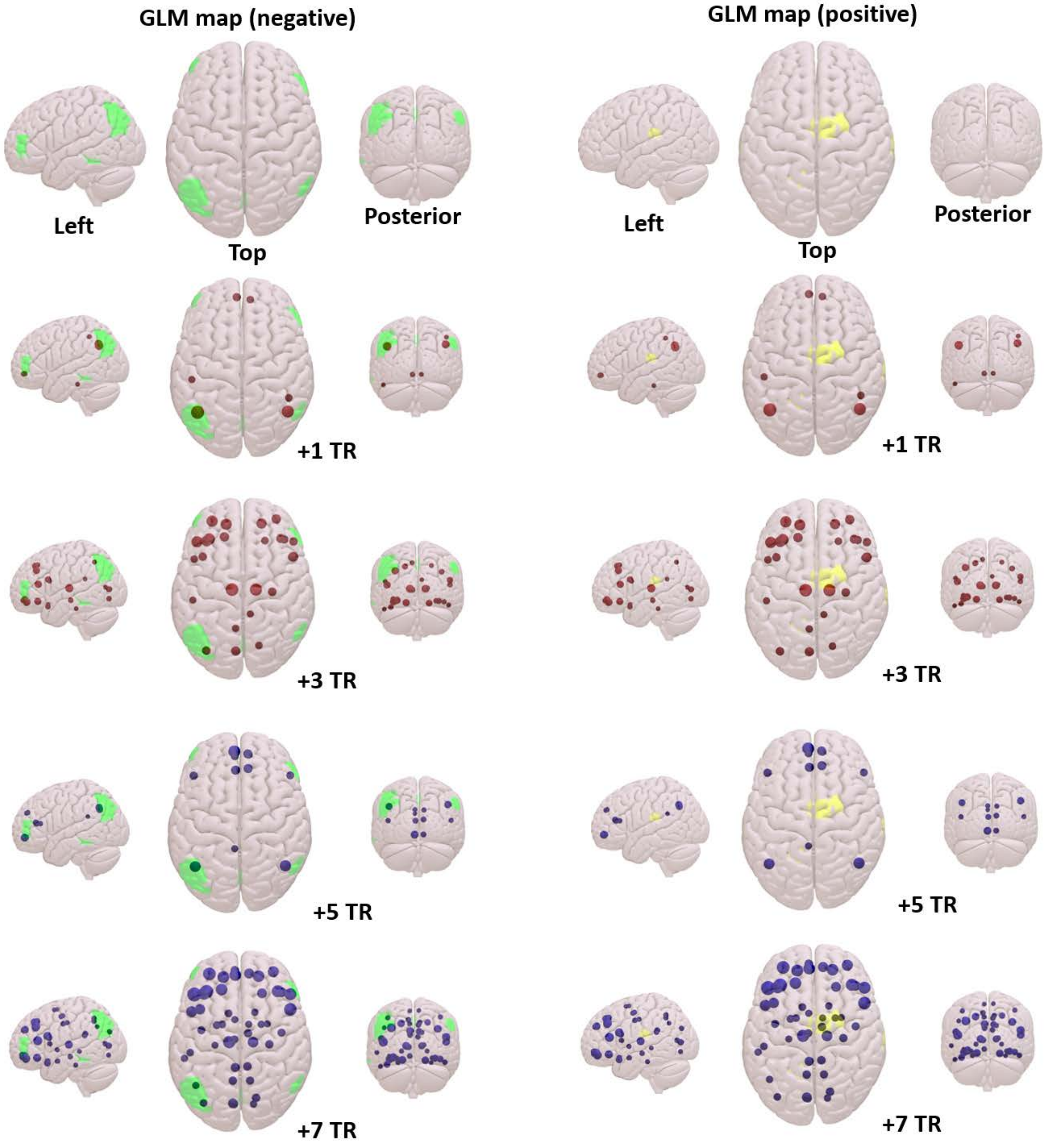

Figure 4: Dynamic BOLD amplitude compared with conventional GLM analysis of the same data. We undertook a conventional GLM analysis of the 95 GSW events analysed in the main manuscript, using SPM8 (further details available from the authors on request). Top left: orthogonal "glass-brain" projections of brain regions (green) showing a BOLD deactivation at the time of GSW onset (*P*<0.001 uncorrected, extent threshold 5 voxels). Top right: orthogonal "glass-brain" projections of brain regions (yellow) showing a BOLD activation at the time of GSW onset (*P*<0.001 uncorrected, extent threshold 5 voxels). We superimpose onto this static map the pattern of BOLD amplitude increases and decreases at TRs +1, +3. +5 and +7 (as shown in Figure 1, main manuscript). Note that cortical regions showing early increase (TR+1) and early decrease (TR+5) in BOLD amplitude coincide closely with GLM deactivations; whereas the thalamic region that shows GLM activation coincides with thalamic increase and decrease in BOLD amplitude that occurs relatively late (increase at TR+3, decrease at TR+7).

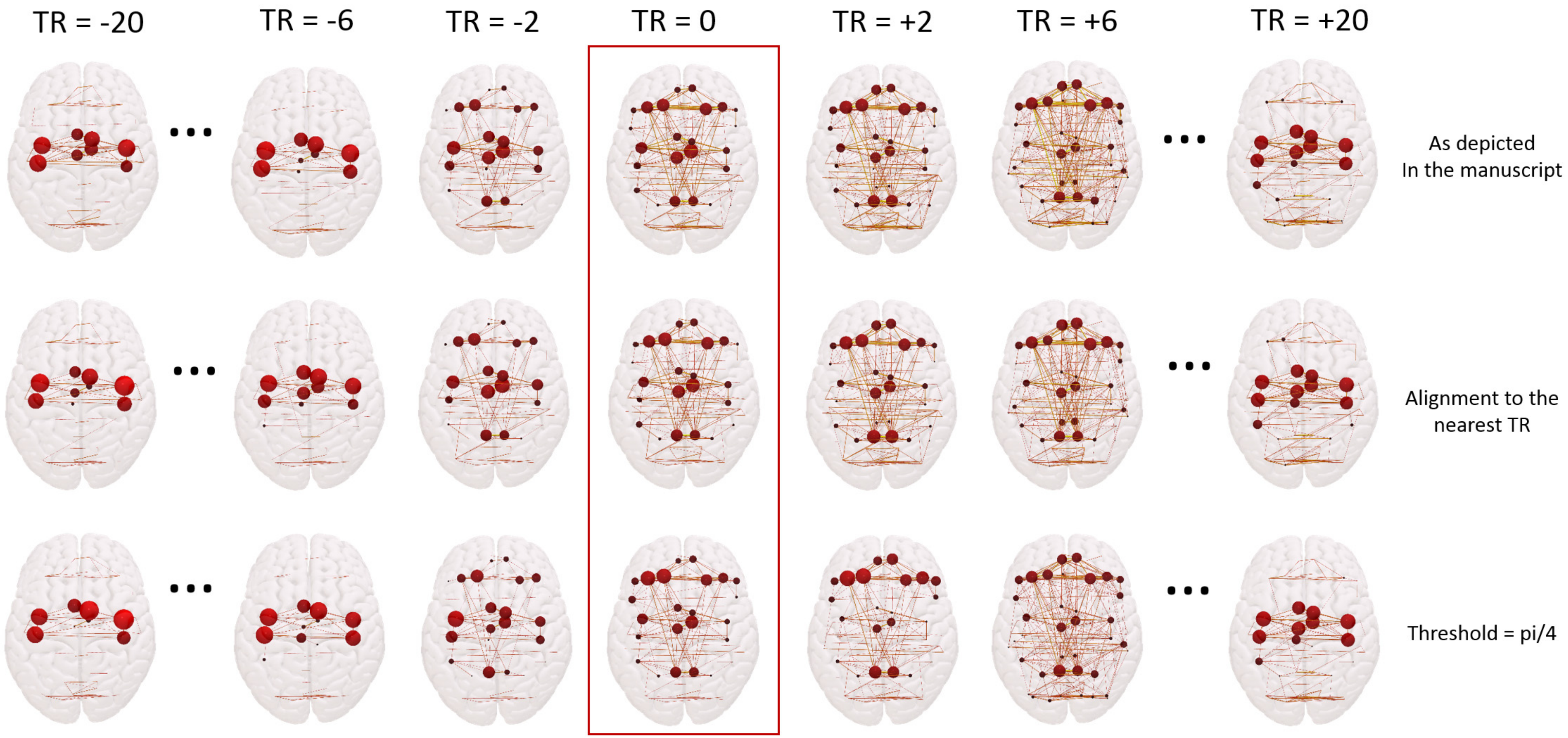

Figure 5: Re-analysis of functional networks using different methodological choices. In the main manuscript, we chose to re-align the GSW onset to the nearest 0.01s, and thresholded phase differences between regions at pi/6 (summarised in the top row of the figure). Here, we illustrate in the middle row the impact of re-aligning GSW onsets to the nearest TR, which avoids any need to re-sample the BOLD timeseries (while maintining a phase difference threshold of pi/6). In the bottom row, we show the effect of using a phase difference threshold of pi/4 (while re-aligning GSW onsets to the nearest 0.01s). Note that the findings are extremely similar despite this range of methodological choices, strongly suggesting our findings are robust to such choices.

Sub	Right pre-central	Left superior medial frontal	Left precuneus	Right superior temporal pole
#1	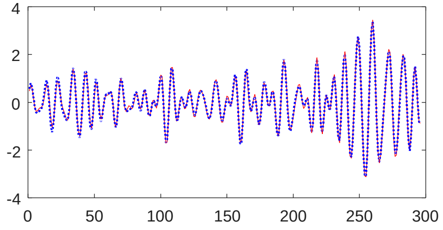	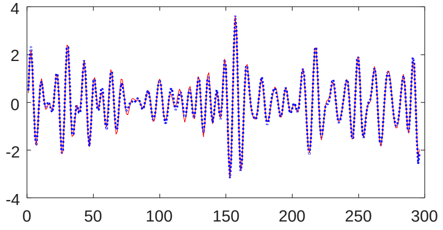	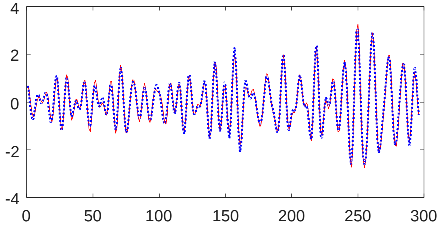	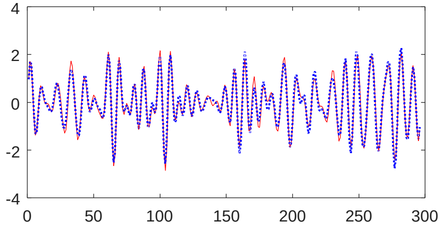
#2	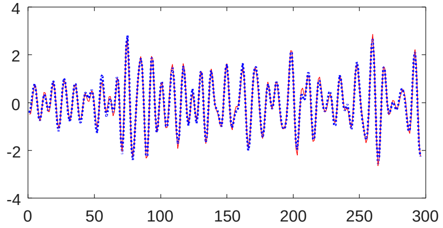	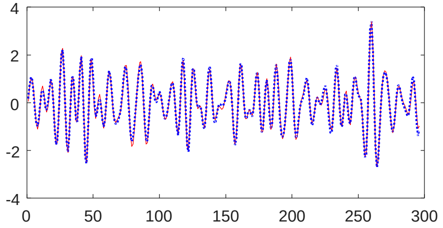	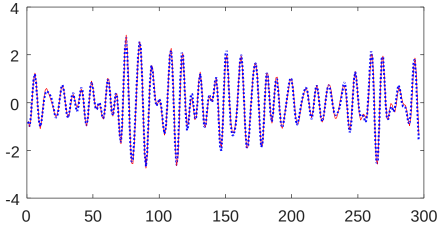	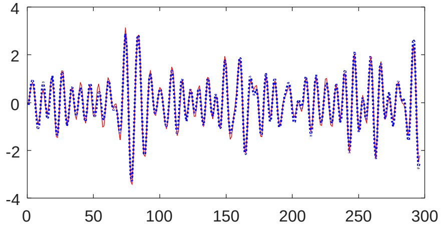
#3	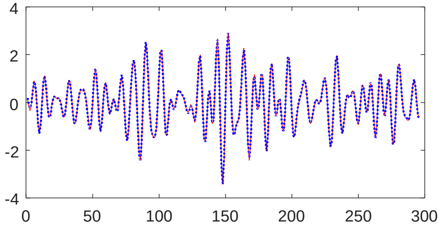	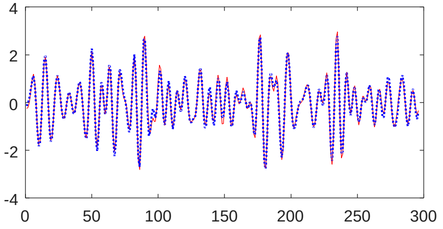	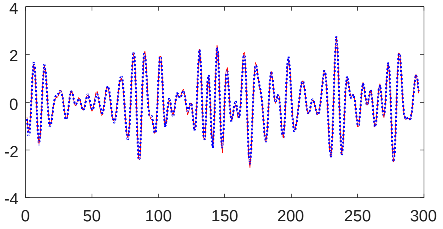	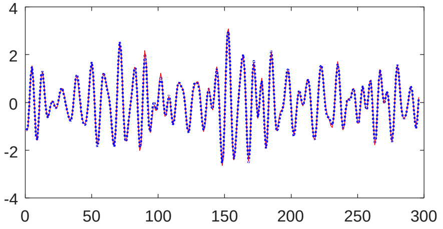
#4	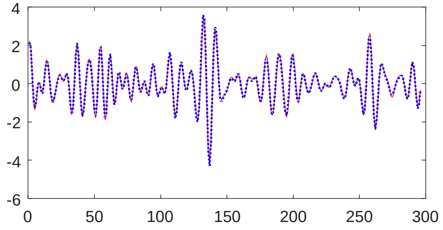	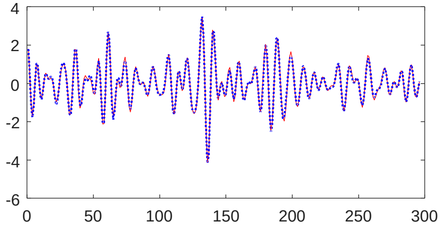	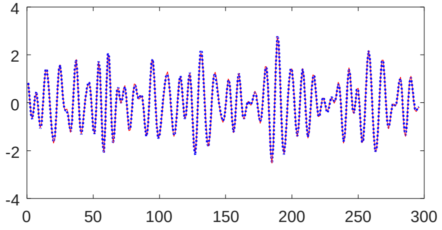	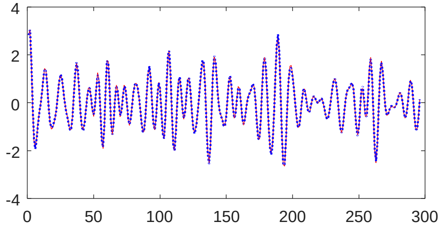

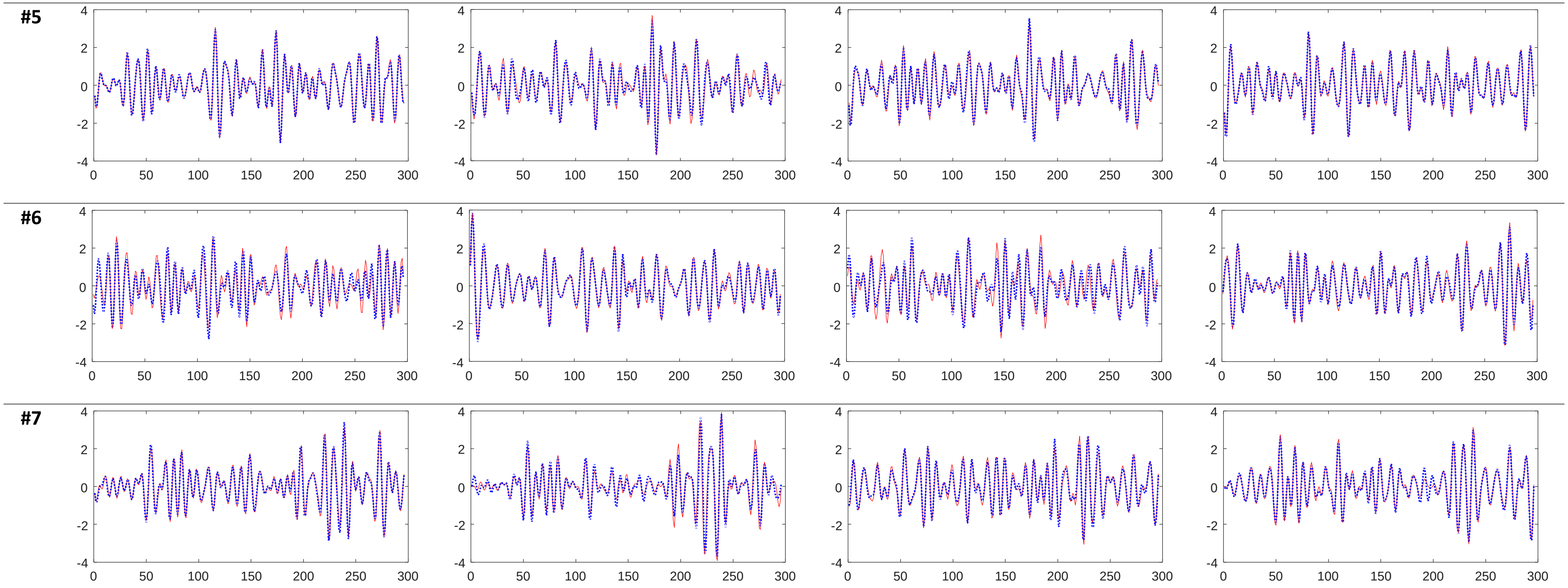

Figure 6: Correlation between mean and first principal component of filtered BOLD signal in each region. We show here filtered BOLD data from four representative regions in 7 representative subjects. In each panel, signal amplitude is shown on the y-axis and time (in intervals of a TR) on the x-axis. The region mean is shown as a blue dotted line and the region first principal component as a red solid line. Note that the first principal component and the mean are extremely highly correlated: across all 90 regions in all 7 subjects, the average correlation coefficient was 0.982 (SD 0.046).

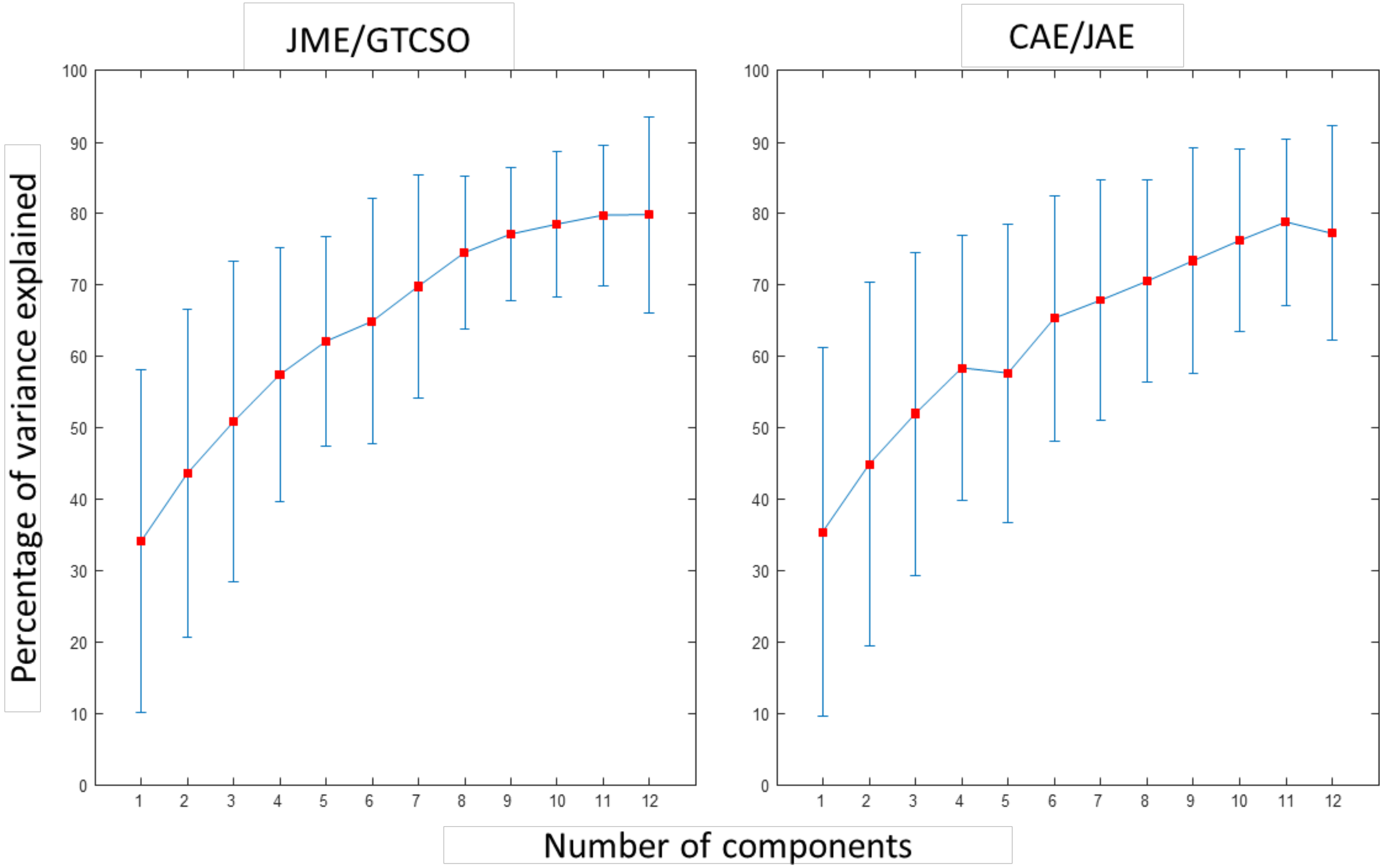

Figure 7: The relationship between the number of components from tensor decomposition used to represent the signal in each region, versus the percentage of explained signal variance in the region. Note a clear trend across subjects that the proportion of explained variance increases asymptotically with increasing number of components, reaching a maximum at 11-12 components; and also that there is a clear trend that the variance across subjects in the explained variance diminishes with increasing components. This observation supports our choice of using 12 tensor components.

Region	TR-11		TR-10		TR-4		TR-3		TR-2		TR-1		TR-0 (GSW)		TR+1 (GSW)		TR+2 (GSW)		TR+3		TR+4		TR+10		TR+11	
	EigC	Zamp	EigC	Zamp	EigC	Zamp	EigC	Zamp	EigC	Zamp	EigC	Zamp	EigC	Zamp	EigC	Zamp	EigC	Zamp	EigC	Zamp	EigC	Zamp	EigC	Zamp	EigC	Zamp
'Precentral_L'	*0.42*	0.00	*0.40*	0.00	*0.44*	0.00	*0.40*	0.00	*0.33*	0.00	*0.30*	0.00	*0.27*	0.00	*0.22*	0.00	*0.22*	**0.36**	*0.16*	0.00	*0.17*	0.00	*0.18*	0.00	*0.18*	0.00
'Precentral_R'	*0.41*	0.00	*0.40*	0.00	*0.37*	0.00	*0.32*	0.00	*0.24*	0.00	*0.20*	0.00	*0.17*	0.00	*0.15*	0.00	*0.15*	0.00	*0.13*	0.00	*0.12*	0.00	*0.13*	0.00	*0.16*	0.00
'Frontal_Sup_L'	0.00	0.00	0.00	0.00	0.00	0.00	*0.16*	0.00	*0.28*	0.00	*0.30*	0.00	*0.31*	0.00	*0.29*	0.00	*0.31*	**0.55**	*0.33*	**0.47**	*0.27*	0.00	*0.27*	0.00	*0.31*	0.00
'Frontal_Sup_R'	0.00	0.00	0.00	0.00	0.00	0.00	*0.08*	0.00	*0.19*	0.00	*0.27*	0.00	*0.29*	0.00	*0.27*	0.00	*0.29*	**0.48**	*0.33*	**0.39**	*0.29*	0.00	*0.32*	0.00	*0.29*	0.00
'Frontal_Sup_Orb_L'	0.00	0.00	0.00	0.00	0.00	0.00	0.00	0.00	0.00	0.00	0.00	0.00	0.00	0.00	0.00	0.00	0.00	**0.59**	0.00	**0.56**	0.00	0.00	0.00	0.00	0.00	0.00
'Frontal_Sup_Orb_R'	0.00	0.00	0.00	0.00	0.00	0.00	0.00	0.00	0.00	0.00	0.00	0.00	0.00	0.00	0.00	0.00	0.00	**0.51**	0.00	**0.51**	0.00	0.00	0.00	0.00	0.00	0.00
'Frontal_Mid_L'	0.00	0.00	0.00	0.00	0.00	0.00	*0.08*	0.00	*0.24*	0.00	*0.28*	0.00	*0.29*	0.00	*0.28*	0.00	*0.32*	**0.61**	*0.33*	**0.57**	*0.30*	0.00	*0.32*	0.00	*0.32*	**0.38**
'Frontal_Mid_R'	0.00	0.00	0.00	0.00	0.00	0.00	*0.06*	0.00	*0.19*	0.00	*0.21*	0.00	*0.24*	0.00	*0.25*	0.00	*0.24*	**0.58**	*0.25*	**0.45**	*0.29*	0.00	*0.28*	0.00	*0.28*	0.00
'Frontal_Mid_Orb_L'	0.00	0.00	0.00	0.00	0.00	0.00	0.00	0.00	0.00	0.00	0.00	0.00	0.00	0.00	0.00	0.00	0.00	**0.70**	0.00	**0.57**	0.00	0.00	0.00	0.00	0.00	0.00
'Frontal_Mid_Orb_R'	0.00	0.00	0.00	0.00	0.00	0.00	0.00	0.00	0.00	0.00	0.00	0.00	0.00	0.00	0.00	0.00	0.00	**0.45**	0.00	**0.47**	0.00	0.00	0.00	0.00	0.00	0.00
'Frontal_Inf_Oper_L'	0.00	0.00	0.00	0.00	0.00	0.00	0.00	0.00	0.00	0.00	*0.05*	0.00	*0.09*	0.00	*0.13*	0.00	*0.11*	**0.46**	*0.07*	**0.39**	*0.07*	0.00	*0.04*	0.00	0.02	**0.38**
'Frontal_Inf_Oper_R'	0.00	0.00	0.00	0.00	0.00	0.00	0.00	0.00	0.00	0.00	0.01	0.00	*0.05*	0.00	*0.08*	0.00	*0.06*	**0.53**	*0.06*	**0.49**	*0.05*	0.00	*0.18*	0.00	*0.14*	**0.37**
'Frontal_Inf_Tri_L'	0.00	0.00	0.00	0.00	0.00	0.00	0.00	0.00	*0.06*	0.00	*0.11*	0.00	*0.14*	0.00	*0.22*	0.00	*0.19*	**0.74**	*0.18*	**0.56**	*0.14*	0.00	*0.13*	0.00	*0.09*	**0.42**
'Frontal_Inf_Tri_R'	0.00	0.00	0.00	0.00	0.00	0.00	0.00	0.00	0.01	0.00	0.03	0.00	*0.10*	0.00	*0.20*	0.00	*0.13*	**0.59**	*0.15*	**0.42**	*0.15*	0.00	*0.18*	0.00	*0.12*	**0.37**
'Frontal_Inf_Orb_L'	0.00	0.00	0.00	0.00	0.00	0.00	0.00	0.00	0.00	0.00	0.00	0.00	0.01	0.00	*0.05*	0.00	0.02	**0.63**	0.02	**0.55**	0.01	0.00	0.02	0.00	0.00	0.00
'Frontal_Inf_Orb_R'	0.00	0.00	0.00	0.00	0.00	0.00	0.00	0.00	0.00	0.00	0.00	0.00	0.01	0.00	*0.07*	0.00	0.01	**0.53**	0.02	**0.41**	0.02	0.00	*0.07*	0.00	0.01	0.00
'Rolandic_Oper_L'	0.01	0.00	0.01	0.00	0.00	0.00	0.00	0.00	0.00	0.00	0.00	0.00	0.00	0.00	0.00	0.00	0.00	0.00	0.00	0.00	0.00	0.00	0.00	0.00	0.00	0.00
'Rolandic_Oper_R'	0.00	0.00	0.00	0.00	0.00	0.00	0.00	0.00	0.00	0.00	0.00	0.00	0.00	0.00	0.00	0.00	0.00	0.00	0.00	0.00	0.00	0.00	0.00	0.00	0.00	0.00
'Supp_Motor_Area_L'	*0.36*	0.00	*0.35*	0.00	*0.36*	0.00	*0.36*	0.00	*0.28*	0.00	*0.24*	0.00	*0.20*	0.00	*0.14*	0.00	*0.15*	0.00	*0.13*	0.00	*0.11*	0.00	*0.07*	0.00	*0.10*	0.00
'Supp_Motor_Area_R'	*0.32*	0.00	*0.36*	0.00	*0.43*	0.00	*0.38*	0.00	*0.29*	0.00	*0.20*	0.00	*0.17*	0.00	*0.12*	0.00	*0.12*	0.00	*0.08*	0.00	*0.09*	0.00	*0.06*	0.00	*0.11*	0.00
'Olfactory_L'	0.00	0.00	0.00	0.00	0.00	0.00	0.00	0.00	0.00	0.00	0.00	0.00	0.00	0.00	0.00	0.00	0.00	0.00	0.00	0.00	0.00	0.00	0.00	0.00	0.00	0.00
'Olfactory_R'	0.00	0.00	0.00	0.00	0.00	0.00	0.00	0.00	0.00	0.00	0.00	0.00	0.00	0.00	0.00	0.00	0.00	0.00	0.00	0.00	0.00	0.00	0.00	0.00	0.00	0.00
'Frontal_Sup_Medial_L'	0.00	0.00	0.00	0.00	0.00	0.00	0.03	0.00	*0.06*	0.00	*0.10*	0.00	*0.15*	0.00	*0.22*	0.00	*0.24*	**0.50**	*0.22*	0.00	*0.25*	0.00	*0.29*	**0.41**	*0.28*	**0.47**
'Frontal_Sup_Medial_R'	0.00	0.00	0.00	0.00	0.00	0.00	0.03	0.00	*0.13*	0.00	*0.12*	0.00	*0.19*	0.00	*0.24*	0.00	*0.21*	**0.49**	*0.26*	0.00	*0.27*	0.00	*0.28*	**0.36**	*0.29*	**0.40**
'Frontal_Mid_Orb_L'	0.00	0.00	0.00	0.00	0.00	0.00	0.00	0.00	0.00	0.00	0.00	0.00	0.00	0.00	0.00	**0.42**	0.00	**0.54**	0.00	0.00	0.00	0.00	0.00	0.00	0.00	0.00
'Frontal_Mid_Orb_R'	0.00	0.00	0.00	0.00	0.00	0.00	0.00	0.00	0.00	0.00	0.00	0.00	0.00	0.00	0.00	**0.38**	0.00	**0.42**	0.00	0.00	0.00	0.00	0.00	0.00	0.00	0.00
'Rectus_L'	0.00	0.00	0.00	0.00	0.00	0.00	0.00	0.00	0.00	0.00	0.00	0.00	0.00	0.00	0.00	0.00	0.00	0.00	0.00	0.00	0.00	0.00	0.00	0.00	0.00	0.00
'Rectus_R'	0.00	0.00	0.00	0.00	0.00	0.00	0.00	0.00	0.00	0.00	0.00	0.00	0.00	0.00	0.00	0.00	0.00	0.00	0.00	0.00	0.00	0.00	0.00	0.00	0.00	0.00
'Insula_L'	0.00	0.00	0.00	0.00	0.00	0.00	0.00	0.00	0.00	0.00	0.00	0.00	0.00	0.00	0.00	0.00	0.00	0.00	0.00	0.00	0.00	0.00	0.00	0.00	0.00	0.00
'Insula_R'	0.00	0.00	0.00	0.00	0.00	0.00	0.00	0.00	0.00	0.00	0.00	0.00	0.00	0.00	0.00	0.00	0.00	0.00	0.00	0.00	0.00	0.00	0.00	0.00	0.00	0.00
'Cingulum_Ant_L'	0.00	0.00	0.00	0.00	0.00	0.00	0.00	0.00	0.00	0.00	0.00	0.00	0.02	0.00	0.04	0.00	0.03	0.00	0.02	0.00	*0.06*	0.00	*0.05*	**0.35**	0.03	0.00
'Cingulum_Ant_R'	0.00	0.00	0.00	0.00	0.00	0.00	0.00	0.00	0.00	0.00	0.00	0.00	0.02	0.00	0.02	0.00	0.02	0.00	0.00	0.00	0.01	0.00	0.01	0.00	0.00	0.00
'Cingulum_Mid_L'	*0.31*	0.00	*0.35*	0.00	*0.14*	0.00	*0.30*	0.00	*0.32*	0.00	*0.33*	0.00	*0.30*	0.00	*0.24*	0.00	*0.26*	**0.36**	*0.24*	0.00	*0.29*	0.00	*0.20*	0.00	*0.23*	0.00
'Cingulum_Mid_R'	*0.26*	0.00	*0.25*	0.00	*0.25*	0.00	*0.32*	0.00	*0.35*	0.00	*0.36*	0.00	*0.35*	0.00	*0.29*	0.00	*0.25*	**0.37**	*0.29*	0.00	*0.27*	0.00	*0.25*	0.00	*0.27*	0.00
'Cingulum_Post_L'	0.00	0.00	0.00	0.00	0.00	0.00	0.00	0.00	0.00	0.00	0.00	0.00	0.00	0.00	0.00	0.00	*0.06*	**0.48**	*0.07*	0.00	*0.13*	0.00	*0.06*	0.00	0.00	0.00
'Cingulum_Post_R'	0.00	0.00	0.00	0.00	0.00	0.00	0.00	0.00	0.00	0.00	0.00	0.00	0.00	0.00	*0.04*	0.00	*0.06*	**0.36**	*0.10*	**0.35**	*0.15*	0.00	*0.08*	0.00	0.03	0.00
'Hippocampus_L'	0.00	0.00	0.00	0.00	0.00	0.00	0.00	0.00	0.00	0.00	0.00	0.00	0.00	0.00	0.00	0.00	0.00	0.00	0.00	0.00	0.00	0.00	0.00	0.00	0.00	0.00
'Hippocampus_R'	0.00	0.00	0.00	0.00	0.00	0.00	0.00	0.00	0.00	0.00	0.00	0.00	0.00	0.00	0.00	0.00	0.00	0.00	0.00	**0.43**	0.00	0.00	0.00	0.00	0.00	0.00
'ParaHippocampal_L'	0.00	0.00	0.00	0.00	0.00	0.00	0.00	0.00	0.00	0.00	0.00	0.00	0.00	0.00	0.00	0.00	0.00	0.00	0.00	0.00	0.00	0.00	0.00	0.00	0.00	0.00
'ParaHippocampal_R'	0.00	0.00	0.00	0.00	0.00	0.00	0.00	0.00	0.00	0.00	0.00	0.00	0.00	0.00	0.00	0.00	0.00	0.00	0.00	0.00	0.00	0.00	0.00	0.00	0.00	0.00
'Amygdala_L'	0.00	0.00	0.00	0.00	0.00	0.00	0.00	0.00	0.00	0.00	0.00	0.00	0.00	0.00	0.00	0.00	0.00	0.00	0.00	0.00	0.00	0.00	0.00	0.00	0.00	0.00
'Amygdala_R'	0.00	0.00	0.00	0.00	0.00	0.00	0.00	0.00	0.00	0.00	0.00	0.00	0.00	0.00	0.00	0.00	0.00	**0.36**	0.00	0.00	0.00	0.00	0.00	0.00	0.00	0.00
'Calcarine_L'	0.00	0.00	0.00	0.00	0.00	0.00	0.00	0.00	0.00	0.00	0.00	0.00	0.00	0.00	0.01	0.00	0.00	**0.43**	0.00	**0.46**	0.00	0.00	0.01	0.00	0.00	0.00
'Calcarine_R'	0.00	0.00	0.00	0.00	0.00	0.00	0.00	0.00	0.00	0.00	0.00	0.00	0.00	0.00	0.01	0.00	0.01	**0.41**	0.00	**0.38**	0.00	0.00	0.01	0.00	0.00	0.00
'Cuneus_L'	0.00	0.00	0.00	0.00	0.00	0.00	0.00	0.00	0.00	0.00	0.00	0.00	0.00	0.00	0.01	0.00	0.01	**0.39**	0.01	0.00	0.01	0.00	0.02	0.00	0.01	0.00

Table 1: List of all AAL regions with eigenvector centrality value (EigC) and Z-score BOLD amplitude (Zamp) at selected time points (TR). For clarity here, eigenvector centrality value above the average across all TRs and regions is highlighted in green. Similarly, z-score BOLD amplitude above the average is highlighted in red.

Region	TR-11		TR-10		TR-4		TR-3		TR-2		TR-1		TR-0 (GSW)		TR+1 (GSW)		TR+2 (GSW)		TR+3		TR+4		TR+10		TR+11	
	EigC	Zamp	EigC	Zamp	EigC	Zamp	EigC	Zamp	EigC	Zamp	EigC	Zamp	EigC	Zamp	EigC	Zamp	EigC	Zamp	EigC	Zamp	EigC	Zamp	EigC	Zamp	EigC	Zamp
'Cuneus_R'	0.00	0.00	0.00	0.00	0.00	0.00	0.00	0.00	0.00	0.00	0.00	0.00	0.00	0.00	0.02	0.00	0.01	0.37	0.01	0.00	0.01	0.00	0.02	0.00	0.01	0.00
'Lingual_L'	0.00	0.00	0.00	0.00	0.00	0.00	0.00	0.00	0.00	0.00	0.00	0.00	0.00	0.00	0.00	0.00	0.00	0.00	0.00	0.00	0.00	0.00	0.01	0.00	0.00	0.00
'Lingual_R'	0.00	0.00	0.00	0.00	0.00	0.00	0.00	0.00	0.00	0.00	0.00	0.00	0.00	0.00	0.01	0.00	0.00	0.00	0.00	0.00	0.00	0.00	0.01	0.00	0.00	0.00
'Occipital_Sup_L'	0.00	0.00	0.00	0.00	0.00	0.00	0.00	0.00	0.00	0.00	0.00	0.00	0.00	0.00	0.03	0.00	0.02	0.00	0.02	0.00	0.02	0.00	0.03	0.00	0.02	0.00
'Occipital_Sup_R'	0.00	0.00	0.00	0.00	0.00	0.00	0.00	0.00	0.00	0.00	0.00	0.00	0.00	0.00	0.04	0.00	0.03	0.00	0.03	0.00	0.04	0.00	0.04	0.00	0.03	0.00
'Occipital_Mid_L'	0.00	0.00	0.00	0.00	0.00	0.00	0.00	0.00	0.00	0.00	0.00	0.00	0.00	0.00	0.03	0.00	0.02	0.00	0.01	0.00	0.02	0.00	0.06	0.00	0.01	0.00
'Occipital_Mid_R'	0.00	0.00	0.00	0.00	0.00	0.00	0.00	0.00	0.00	0.00	0.00	0.00	0.00	0.00	0.02	0.00	0.01	0.00	0.01	0.00	0.01	0.00	0.06	0.00	0.01	0.00
'Occipital_Inf_L'	0.00	0.00	0.00	0.00	0.00	0.00	0.00	0.00	0.00	0.00	0.00	0.00	0.00	0.00	0.02	0.00	0.01	0.46	0.01	0.43	0.01	0.00	0.02	0.00	0.01	0.00
'Occipital_Inf_R'	0.00	0.00	0.00	0.00	0.00	0.00	0.00	0.00	0.00	0.00	0.00	0.00	0.00	0.00	0.01	0.00	0.01	0.00	0.00	0.00	0.01	0.00	0.02	0.00	0.00	0.00
'Fusiform_L'	0.00	0.00	0.00	0.00	0.00	0.00	0.00	0.00	0.00	0.00	0.00	0.00	0.00	0.00	0.01	0.00	0.00	0.37	0.00	0.00	0.01	0.00	0.00	0.00	0.00	0.00
'Fusiform_R'	0.00	0.00	0.00	0.00	0.00	0.00	0.00	0.00	0.00	0.00	0.00	0.00	0.00	0.00	0.01	0.00	0.00	0.00	0.00	0.00	0.01	0.00	0.01	0.00	0.00	0.00
'Postcentral_L'	0.40	0.00	0.39	0.00	0.39	0.00	0.33	0.00	0.24	0.00	0.19	0.00	0.16	0.00	0.09	0.00	0.10	0.00	0.03	0.00	0.05	0.00	0.05	0.00	0.08	0.00
'Postcentral_R'	0.29	0.00	0.28	0.00	0.31	0.00	0.27	0.00	0.19	0.00	0.19	0.00	0.16	0.00	0.13	0.00	0.11	0.00	0.05	0.00	0.07	0.00	0.04	0.00	0.06	0.00
'Parietal_Sup_L'	0.00	0.00	0.00	0.00	0.00	0.00	0.01	0.00	0.02	0.00	0.08	0.00	0.07	0.00	0.13	0.00	0.12	0.36	0.10	0.00	0.10	0.00	0.10	0.00	0.10	0.00
'Parietal_Sup_R'	0.00	0.00	0.00	0.00	0.00	0.00	0.00	0.00	0.04	0.00	0.06	0.00	0.07	0.00	0.11	0.00	0.10	0.00	0.10	0.00	0.15	0.00	0.14	0.00	0.10	0.00
'Parietal_Inf_L'	0.07	0.00	0.00	0.00	0.08	0.00	0.06	0.00	0.08	0.00	0.16	0.00	0.13	0.00	0.13	0.00	0.12	0.00	0.06	0.00	0.12	0.00	0.06	0.00	0.04	0.00
'Parietal_Inf_R'	0.01	0.00	0.00	0.00	0.01	0.00	0.01	0.00	0.02	0.00	0.03	0.00	0.03	0.00	0.08	0.38	0.04	0.00	0.03	0.00	0.04	0.00	0.02	0.00	0.00	0.00
'SupraMarginal_L'	0.00	0.00	0.00	0.00	0.00	0.00	0.00	0.00	0.00	0.00	0.00	0.00	0.00	0.00	0.00	0.00	0.00	0.00	0.00	0.00	0.00	0.00	0.00	0.00	0.00	0.00
'SupraMarginal_R'	0.00	0.00	0.00	0.00	0.00	0.00	0.00	0.00	0.00	0.00	0.00	0.00	0.00	0.00	0.01	0.00	0.00	0.00	0.00	0.00	0.00	0.00	0.00	0.00	0.00	0.00
'Angular_L'	0.00	0.00	0.00	0.00	0.00	0.00	0.00	0.00	0.00	0.00	0.00	0.00	0.00	0.44	0.00	0.66	0.01	0.42	0.00	0.00	0.01	0.00	0.00	0.40	0.00	0.00
'Angular_R'	0.00	0.00	0.00	0.00	0.00	0.00	0.00	0.00	0.00	0.00	0.00	0.00	0.02	0.00	0.05	0.61	0.06	0.41	0.03	0.00	0.04	0.00	0.02	0.38	0.03	0.00
'Precuneus_L'	0.00	0.00	0.00	0.00	0.00	0.00	0.16	0.00	0.26	0.00	0.24	0.00	0.26	0.00	0.27	0.00	0.29	0.41	0.31	0.38	0.28	0.00	0.30	0.00	0.31	0.00
'Precuneus_R'	0.00	0.00	0.00	0.00	0.00	0.00	0.06	0.00	0.22	0.00	0.22	0.00	0.24	0.00	0.26	0.00	0.29	0.36	0.28	0.00	0.27	0.00	0.28	0.00	0.31	0.00
'Paracentral_Lobule_L'	0.11	0.00	0.11	0.00	0.15	0.00	0.10	0.00	0.07	0.00	0.04	0.00	0.03	0.00	0.05	0.00	0.04	0.00	0.00	0.00	0.00	0.00	0.00	0.00	0.03	0.00
'Paracentral_Lobule_R'	0.00	0.00	0.00	0.00	0.00	0.00	0.00	0.00	0.00	0.00	0.00	0.00	0.00	0.00	0.00	0.00	0.00	0.00	0.00	0.00	0.00	0.00	0.00	0.00	0.00	0.00
'Caudate_L'	0.00	0.00	0.00	0.00	0.00	0.00	0.00	0.00	0.00	0.00	0.00	0.00	0.00	0.00	0.00	0.00	0.00	0.00	0.00	0.00	0.00	0.00	0.00	0.00	0.00	0.00
'Caudate_R'	0.00	0.00	0.00	0.00	0.00	0.00	0.00	0.00	0.00	0.00	0.00	0.00	0.00	0.00	0.00	0.00	0.00	0.00	0.00	0.00	0.00	0.00	0.00	0.00	0.00	0.00
'Putamen_L'	0.00	0.00	0.00	0.00	0.00	0.00	0.00	0.00	0.00	0.00	0.00	0.00	0.00	0.00	0.00	0.00	0.00	0.00	0.00	0.00	0.00	0.00	0.00	0.00	0.00	0.00
'Putamen_R'	0.00	0.00	0.00	0.00	0.00	0.00	0.00	0.00	0.00	0.00	0.00	0.00	0.00	0.00	0.00	0.00	0.00	0.00	0.00	0.00	0.00	0.00	0.00	0.00	0.00	0.00
'Pallidum_L'	0.00	0.00	0.00	0.00	0.00	0.00	0.00	0.00	0.00	0.00	0.00	0.00	0.00	0.00	0.00	0.00	0.00	0.00	0.00	0.00	0.00	0.00	0.00	0.40	0.00	0.00
'Pallidum_R'	0.00	0.00	0.00	0.00	0.00	0.00	0.00	0.00	0.00	0.00	0.00	0.00	0.00	0.00	0.00	0.00	0.00	0.00	0.00	0.00	0.00	0.00	0.00	0.37	0.00	0.00
'Thalamus_L'	0.00	0.00	0.00	0.00	0.00	0.00	0.00	0.00	0.00	0.00	0.00	0.00	0.00	0.00	0.00	0.00	0.00	0.38	0.00	0.64	0.00	0.55	0.00	0.00	0.00	0.00
'Thalamus_R'	0.00	0.00	0.00	0.00	0.00	0.00	0.00	0.00	0.00	0.00	0.00	0.00	0.00	0.00	0.00	0.00	0.00	0.00	0.00	0.61	0.00	0.59	0.00	0.00	0.00	0.00
'Heschl_L'	0.00	0.00	0.00	0.00	0.00	0.00	0.00	0.00	0.00	0.00	0.00	0.00	0.00	0.00	0.00	0.00	0.00	0.00	0.00	0.00	0.00	0.00	0.00	0.00	0.00	0.00
'Heschl_R'	0.00	0.00	0.00	0.00	0.00	0.00	0.00	0.00	0.00	0.00	0.00	0.00	0.00	0.00	0.00	0.00	0.00	0.00	0.00	0.00	0.00	0.00	0.00	0.00	0.00	0.00
'Temporal_Sup_L'	0.01	0.00	0.01	0.00	0.00	0.00	0.00	0.00	0.00	0.00	0.00	0.00	0.00	0.00	0.00	0.00	0.00	0.00	0.00	0.00	0.00	0.00	0.00	0.00	0.00	0.00
'Temporal_Sup_R'	0.07	0.00	0.06	0.00	0.00	0.00	0.00	0.00	0.00	0.00	0.00	0.00	0.00	0.00	0.00	0.00	0.00	0.00	0.00	0.00	0.00	0.00	0.00	0.00	0.00	0.00
'Temporal_Pole_Sup_L'	0.00	0.00	0.00	0.00	0.00	0.00	0.00	0.00	0.00	0.00	0.00	0.00	0.00	0.00	0.00	0.00	0.00	0.50	0.00	0.43	0.00	0.00	0.00	0.00	0.00	0.00
'Temporal_Pole_Sup_R'	0.00	0.00	0.00	0.00	0.00	0.00	0.00	0.00	0.00	0.00	0.00	0.00	0.00	0.00	0.01	0.00	0.00	0.49	0.00	0.37	0.00	0.00	0.00	0.00	0.00	0.00
'Temporal_Mid_L'	0.00	0.00	0.00	0.00	0.00	0.00	0.00	0.00	0.00	0.00	0.00	0.00	0.00	0.00	0.02	0.00	0.00	0.00	0.00	0.00	0.01	0.00	0.00	0.00	0.00	0.00
'Temporal_Mid_R'	0.00	0.00	0.00	0.00	0.00	0.00	0.00	0.00	0.00	0.00	0.00	0.00	0.00	0.00	0.02	0.00	0.00	0.00	0.00	0.00	0.02	0.00	0.04	0.00	0.03	0.00
'Temporal_Pole_Mid_L'	0.00	0.00	0.00	0.00	0.00	0.00	0.00	0.00	0.00	0.00	0.00	0.00	0.00	0.00	0.00	0.00	0.00	0.00	0.00	0.00	0.00	0.00	0.00	0.00	0.00	0.00
'Temporal_Pole_Mid_R'	0.00	0.00	0.00	0.00	0.00	0.00	0.00	0.00	0.00	0.00	0.00	0.00	0.00	0.00	0.00	0.00	0.00	0.00	0.00	0.00	0.00	0.00	0.00	0.00	0.00	0.00
'Temporal_Inf_L'	0.00	0.00	0.00	0.00	0.00	0.00	0.00	0.00	0.00	0.00	0.00	0.00	0.00	0.00	0.01	0.36	0.00	0.41	0.00	0.36	0.00	0.00	0.01	0.00	0.00	0.00
'Temporal_Inf_R'	0.00	0.00	0.00	0.00	0.00	0.00	0.00	0.00	0.00	0.00	0.00	0.00	0.00	0.00	0.03	0.00	0.01	0.37	0.01	0.00	0.05	0.00	0.03	0.00	0.00	0.00

Table 1 (cont.): List of all AAL regions with eigenvector centrality value (EigC) and Z-score BOLD amplitude (Zamp) at selected time points (TR). For clarity here, eigenvector centrality value above the average across all TRs and regions is highlighted in green. Similarly, z-score BOLD amplitude above the average is highlighted in red.

GSW	Sensorimotor	Occipital
'Precentral_L'	'Precentral_L'	'Calcarine_L'
'Precentral_R'	'Precentral_R'	'Calcarine_R'
'Frontal_Sup_L'	'Supp_Motor_Area_L'	'Cuneus_L'
'Frontal_Sup_R'	'Supp_Motor_Area_R'	'Cuneus_R'
'Frontal_Mid_L'	'Cingulum_Mid_L'	'Lingual_L'
'Frontal_Mid_R'	'Cingulum_Mid_R'	'Lingual_R'
'Frontal_Inf_Tri_L'	'Postcentral_L'	'Occipital_Sup_L'
'Frontal_Inf_Tri_R'	'Postcentral_R'	'Occipital_Sup_R'
'Supp_Motor_Area_L'		'Occipital_Mid_L'
'Frontal_Sup_Medial_L'		'Occipital_Mid_R'
'Frontal_Sup_Medial_R'		'Occipital_Inf_L'
'Cingulum_Mid_L'		'Occipital_Inf_R'
'Cingulum_Mid_R'		'Fusiform_R'
'Precuneus_L'		
'Precuneus_R'		

Table 2: Three canonical networks (GSW, Sensorimotor, and Occipital networks) and their nodes.
